# Inclusion complexes of 2-methoxyestradiol with dimethylated and permethylated β-cyclodextrins: models for cyclodextrin–steroid interaction

**DOI:** 10.3762/bjoc.11.281

**Published:** 2015-12-16

**Authors:** Mino R Caira, Susan A Bourne, Halima Samsodien, Vincent J Smith

**Affiliations:** 1 Centre for Supramolecular Chemistry Research (CSCR), Department of Chemistry, University of Cape Town, Rondebosch 7701, South Africa

**Keywords:** complexation, cyclodextrin, 2-methoxyestradiol, solubility, X-ray diffraction

## Abstract

The interaction between the potent anticancer agent 2-methoxyestradiol (2ME) and a series of cyclodextrins (CDs) was investigated in the solid state using thermal analysis and X-ray diffraction, while the possibility of enhancing its poor aqueous solubility with CDs was probed by means of equilibrium solubility and dissolution rate measurements. Single crystal X-ray diffraction studies of the inclusion complexes between 2ME and the derivatised cyclodextrins heptakis(2,6-di-*O*-methyl)-β-CD (DIMEB) and heptakis(2,3,6-tri-*O*-methyl)-β-CD (TRIMEB) revealed for the first time the nature of the encapsulation of a bioactive steroid by representative CD host molecules. Inclusion complexation invariably involves insertion of the D-ring of 2ME from the secondary side of each CD molecule, with the 17-OH group generally hydrogen bonding to a host glycosidic oxygen atom within the CD cavity, while the A-ring and part of the B-ring of 2ME protrude from the secondary side. In the case of the TRIMEB·2ME complex, there is evidence that complexation proceeds with mutual conformational adaptation of host and guest molecules. The aqueous solubility of 2ME was significantly enhanced by CDs, with DIMEB, TRIMEB, randomly methylated β-CD and hydroxypropyl-β-CD being the most effective hosts. The 2:1 host–guest β-CD inclusion complex, prepared by two methods, yielded very rapid dissolution in water at 37 °C relative to untreated 2ME, attaining complete dissolution within 15 minutes (co-precipitated complex) and 45 minutes (complex from kneading).

## Introduction

**Figure 1:**
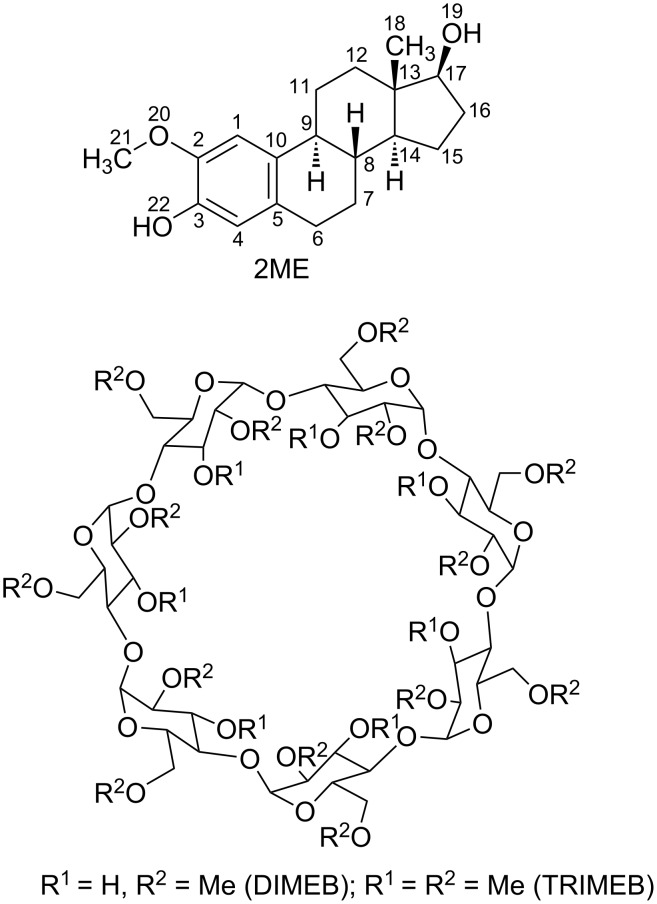
Chemical structures of 2-methoxyestradiol (top) and the derivatised CDs DIMEB and TRIMEB (bottom).

This report focuses on the modes of inclusion of the anticancer agent 2-methoxyestradiol (2ME, Figure 1) in the host cyclodextrins (CDs) heptakis(2,6-di-*O*-methyl)-β-CD (DIMEB) and heptakis(2,3,6-tri-*O*-methyl)-β-CD (TRIMEB) in the solid state. The structures to be described are the first for crystalline CD inclusion complexes of a representative bioactive steroid and as such, their molecular structures shed some light on the nature of cyclodextrin-steroid interactions, including the phenomenon of ‘mutually induced fit’ accompanying complexation. This feature, while commonly observed in protein–ligand recognition and selection, is rare for small molecule structures [1]. In addition to the solid-state results detailed below, we report original data on the use of CDs to enhance the aqeous solubility of 2ME.

The title steroid, originally identified as an endogenous metabolite of the most common estrogen hormone, 17β-estradiol, has been intensively studied owing to its proven potent antiangiogenic, antiproliferative and antitumoral activities [2–3]. However, its poor bioavailability has retarded its development as a drug [4–5]. Although recent reports of ongoing clinical trials with 2ME continue to appear [6–7], attention has shifted towards its more promising derivatives, such as 2-methoxyestradiol-3,17-di,*O*-bis-sulfamate (2MES), with higher potency and improved pharmacokinetic properties [8]. Nevertheless, as noted in our recent report on some aspects of the solid-state chemistry of 2ME and 2MES [9] several new indications for 2ME in the area of anticancer therapy, in particular, coupled with the well-established low toxicity of underivatised 2ME, have revived interest in this compound [9–10], providing ongoing motivation for increasing its poor bioavailability.

We therefore embarked on a study of 2ME [11] with the objectives of investigating both the interactions between CD molecules and 2ME in the solid-state as well as the effects of CDs on the aqueous solubility of 2ME, neither of these aspects having been given significant attention in the literature. Encapsulation of poorly soluble drug molecules by CDs with the aim of enhancing drug bioavailability has been practised for over four decades, the first demonstration of both in vivo and in vitro effects of complexation on drug release having been reported in the early 1970s [12–13]. Steroid hormones were among the first bioactive compounds whose aqueous solubilities were shown to be significantly enhanced via CD inclusion. For example, in an early study by Uekama et al. [14], the interactions between 18 steroid hormones and the native cyclodextrins α-, β- and γ-CD in aqueous solution were investigated to gain insight into CD–steroid interaction modes and the stability of the resulting inclusion complexes. These researchers also used Fourier transform infrared spectroscopy (FTIR), thermal analysis and powder X-ray diffraction (PXRD) to infer the existence of solid native CD–steroid inclusion complexes, some of them having host–guest stoichiometric ratios of 2:1 and 3:2 with β-CD and γ-CD, respectively. An early attempt to acquire basic structural information from PXRD data for crystalline inclusion complexes of β-CD with a series of pregnanes as guests led to the estimation of crystal unit cell parameters [15], from which the authors inferred a ‘channel cyclindrical structure’ as a possible arrangement in the crystalline state. However, no definitive structural studies were subsequently published. Our search in the Cambridge Structural Database [16] for CD complex structures revealed two reported crystal structures containing the rocuronium ion, a well-known neuromuscular blocking agent consisting of a steroid nucleus that is highly functionalised [17], this species being encapsulated by specially designed CD derivatives [per-6-deoxy-per-6-carboxyethylsulfanyl-γ-CD and 6-perdeoxy-6-per(4-carboxylatophenyl)thio-γ-CD] capable of accommodating such a large guest species. While being of significant interest in the context of modern anaesthesia [18], this more exotic supramolecular construct does not serve to illustrate CD–steroidal interactions that might be associated with the vast majority of bioactive steroids and our search further revealed that no representative CD–steroid structures containing more common CDs and steroidal guests (e.g., cortisone, prednisolone, estradiol, or their derivatives) have been reported hitherto. Very recently, an account of the crystal structure of a host comprising a γ-CD duplex system connected by two disulfide bonds that forms stable complexes with steroids appeared [19]. However, no crystal structure of any of its inclusion complexes was reported.

Finally, the lack of published studies on the potential for CDs to improve the aqueous solubility of 2ME also prompted our study of this pharmaceutically relevant aspect of the bioactive compound. Here, we report original data that reflect significant solubility enhancements for 2ME effected by a range of CDs as well as comparative dissolution data in aqueous medium to highlight the potential advantages for further development of 2ME.

## Results and Discussion

### Interaction between 2ME and native CDs

Initial studies focused on possible isolation of solid inclusion complexes of 2ME with the native cyclodextrins α-, β- and γ-CD. Following neat co-grinding of each CD and 2ME (1 h, 1:1 molar ratios) and kneading (1 h with water as medium, CD-2ME ratios 1:1 and 2:1), PXRD traces of the products were recorded. These revealed that physical mixtures resulted in all cases but one, namely kneading β-CD and 2ME in a 2:1 molar ratio, where inclusion complexation definitely occurred. This was deduced by comparing the experimental PXRD trace of the product with that of an isostructural inclusion complex, namely the β-CD complex of 4-*tert*-butylbenzyl alcohol (CSD refcode KOFJEU [16]), and finding a reasonable match [20]. This procedure also enabled correct prediction of the space group (*C*222_1_) of the complex and the approximate unit cell dimensions (*a* ≈ 19.2, *b* ≈ 24.4, *c* ≈ 32.8 Å), results which were subsequently confirmed by a single crystal X-ray diffraction study of the same phase obtained via the co-precipitation method. (Relevant PXRD traces and single crystal data are provided in Supporting Information File 1). Further routine characterization of the β-CD complex of 2ME by UV spectrophotometry (at λ = 198.5 nm in MeOH/water 50:50 v/v) coupled with thermogravimetric anlysis (TGA) data (8.8 ± 0.4% one-step mass loss in the range 30–150 °C, *n* = 3) yielded the host–guest stoichiometry (2:1) and crystal water content (12.6 ± 0.6 water molecules per complex unit) respectively, and hence the complex formula (β-CD)_2_·2ME·(12.6 ± 0.6)H_2_O. X-ray crystal structure solution by isomorphous replacement, using as trial model the host molecule coordinates of KOFJEU, led to successful refinement of the host framework but the included 2ME molecule, located within the ‘cage’ provided by the well known hydrogen-bonded β-CD dimeric unit, was severely disordered and no details of its mode of inclusion could be deduced from difference electron-density syntheses. However, despite this deficiency, the inclusion complex was chemically well-defined and hence met the criterion for subsequent solubility studies.

### Interaction between 2ME and the amorphous CD derivatives RAMEB and HPBCD

PXRD, differential scanning calorimetry (DSC) and FTIR methods were employed to investigate the products of kneading between crystalline 2ME and these amorphous host compounds. (Relevant PXRD traces, DSC traces and FTIR spectra are provided in the Supporting Information File 1). In each case, kneading an equimolar physical mixture of the respective host and 2ME produced an amorphous phase, as evidenced by a PXRD pattern devoid of peaks characteristic of the crystalline component 2ME. Consistent with this, no melting endotherm for 2ME was observed in DSC traces of these preparations, whereas the traces of the corresponding CD-2ME physical mixtures revealed fusion of 2ME at a peak temperature of 188 °C (reported mp 187 °C [9]). Definitive inclusion complex formation between 2ME and both of the host molecules was finally confirmed by comparing the FTIR spectrum of pure 2ME with those of the pure CD and the putative CD-2ME inclusion complexes prepared by kneading; characteristic bands for 2ME (at 1600, 2861, 2903, 3179 and 3413 cm^−1^) were seen to either undergo significant shifts or be masked by broad bands of the hosts RAMEB and HPBCD in the spectra of the materials prepared by kneading.

### Preparation and preliminary characterization of crystalline inclusion complexes between 2ME and the hosts DIMEB and TRIMEB

Hot stage microscopy (HSM) was employed for preliminary investigation of single crystals of a putative inclusion complex between 2ME and DIMEB, prepared using the co-precipitation method. (Details of the thermal analysis of the DIMEB complex of 2ME are provided in the Supporting Information File 1). The crystals, immersed in silicone oil and heated from 30 °C at 10 K/min, were initially clear. Bubbles appearing at ≈80 °C indicated commencement of the release of included water. This process appeared to be continuous, posing difficulty in estimating the temperature corresponding to complete dehydration. The anhydrous phase began to discolour at ≈290 °C, indicating the onset of slow decomposition. Vigorous bubbling and the dark brown colour that developed at ≈400 °C signified the onset of rapid final complex decomposition. Corresponding to these events, the DSC trace displayed an endotherm for dehydration in the approximate range 54–105 °C (peak temperature 80 °C), and a sequence of two small endotherms, a large exotherm, and a small endotherm, all of them spanning the range ≈255–350 °C, attributed to final dehydration and the onset of complex decomposition. The absence of any endothermic effect for the fusion of pure 2ME was consistent with inclusion complex formation. A PXRD trace of the material did not match any of those generated for known polymorphs of the pure host DIMEB [16], again supporting the formation of an inclusion complex between DIMEB and 2ME.

As for the characterization of the β-CD·2ME complex described above, single crystals of the putative DIMEB complex were dissolved in a MeOH/water medium and UV spectrophotometric analysis yielded a host–guest ratio of 1:1. Together with the TGA estimate of water content (5.3 ± 0.3%, *n* = 3), the complex formula DIMEB·2ME·(5.5 ± 0.3)H_2_O was indicated.

A putative inclusion complex between 2ME and the fully methylated host TRIMEB was prepared by the co-precipitation method and initially examined by HSM. (Details of the thermal analysis of the TRIMEB complex of 2ME are provided in Supporting Information File 1). Upon heating the crystals under silicone oil, no dehydration was evident and the material melted at 170 °C. Decomposition, indicated by the molten complex turning brown, commenced at ≈350 °C. The melting point in this case is significantly higher than those reported for the pure host TRIMEB in different crystal forms (148 and 157 °C [21]), supporting the presence of a complex between 2ME and TRIMEB. PXRD subsequently confirmed the presence of a new crystalline phase. TGA indicated no significant mass loss over the interval 30–260 °C while the DSC trace displayed only an endotherm of fusion with a peak temperature of 170 °C. Finally, a 1:1 TRIMEB/2ME complex ratio was indicated from UV spectrophotometric measurements of a solution of dissolved single crystals.

### Definitive structural information for crystalline inclusion complexes between 2ME and the hosts DIMEB and TRIMEB

Since the unit cell dimensions of the two inclusion complexes had no counterparts in the Cambridge Crystallographic Database [16], structure solution by isomorphous replacement was not possible. Both crystal structures were therefore solved ab initio by direct methods using the program SHELXD [22]. Model development involved successive difference Fourier syntheses and iterative refinement by the full-matrix least-squares technique using SHELXH [22]. With only a single complex unit DIMEB·2ME in the crystal asymmetric unit, structure solution was fairly straightforward. However, the structure determination and refinement of the TRIMEB inclusion complex proved to be considerably more challenging due to the presence of four TRIMEB·2ME units (A–D) in the asymmetric unit. Table 1 summarises the crystal data and refinement details for the two complexes.

**Table 1:** Crystal data, data collection parameters and refinement details.

abbreviated formulae	DIMEB·2ME·5.3H_2_O	TRIMEB·2ME

complex formula	C_56_H_98_O_35_·C_19_H_26_O_3_·5.3H_2_O	C_63_H_112_O_35_·C_19_H_26_O_3_
formula wt. / g mol^−1^	1729.23	1731.92
crystal system	monoclinic	triclinic
space group	*P*2_1_	*P*1
*a* / (Å)	13.5514(1)	14.1675(2)
*b* / (Å)	24.3921(2)	23.7963(3)
*c* / (Å)	13.7237(1)	28.2467(4)
α / (°)	90.0	85.380(1)
β / (°)	97.047(1)	86.395(1)
γ / (°)	90.0	73.836(1)
*V* / (Å^3^)	4502.06(6)	9108.6(2)
*Z*	2	4
*D_c_* / (Mg m^−3^)	1.276	1.263
μ (MoKα) / (mm^−1^)	0.105	0.099
F (000)	1862	3736
data collection temp. / K	173(2)	173(2)
crystal size / (mm)	0.22 × 0.29 × 0.32	0.18 × 0.23 × 0.26
range scanned θ / °	1.00 – 25.35	1.00 – 24.71
index ranges ±*h,* ±*k,* ±*l*	−16:16; −29:28; −16:16	−16:16; −28:28; −33:33
reflections (total)	15799	62723
independent reflections	15799	62722
reflections with I > 2σ(I)	13657	38538
completeness (%)	99.0	98.8
no. of parameters	1088	3654
goodness-of-fit, *S*	1.028	1.023
R_1_ [*I* > 2σ(*I*)]	0.0817	0.0853
*wR* on *F*^2^	0.2266	0.2324
weighting scheme *a*, *b* in *w* = 1/[σ^2^(F_o_^2^) + (aP)^2^ + (bP)]	0.1665 , 2.8047	0.0960, 16.0732
(Δ/σ)_mean_	< 0.001	0.001
Δρ excursions / e Å^−3^	−0.63 and 0.85	−0.42 and 0.83
CCDC deposition no.	CCDC 1428344	CCDC 1428345

**Figure 2:**
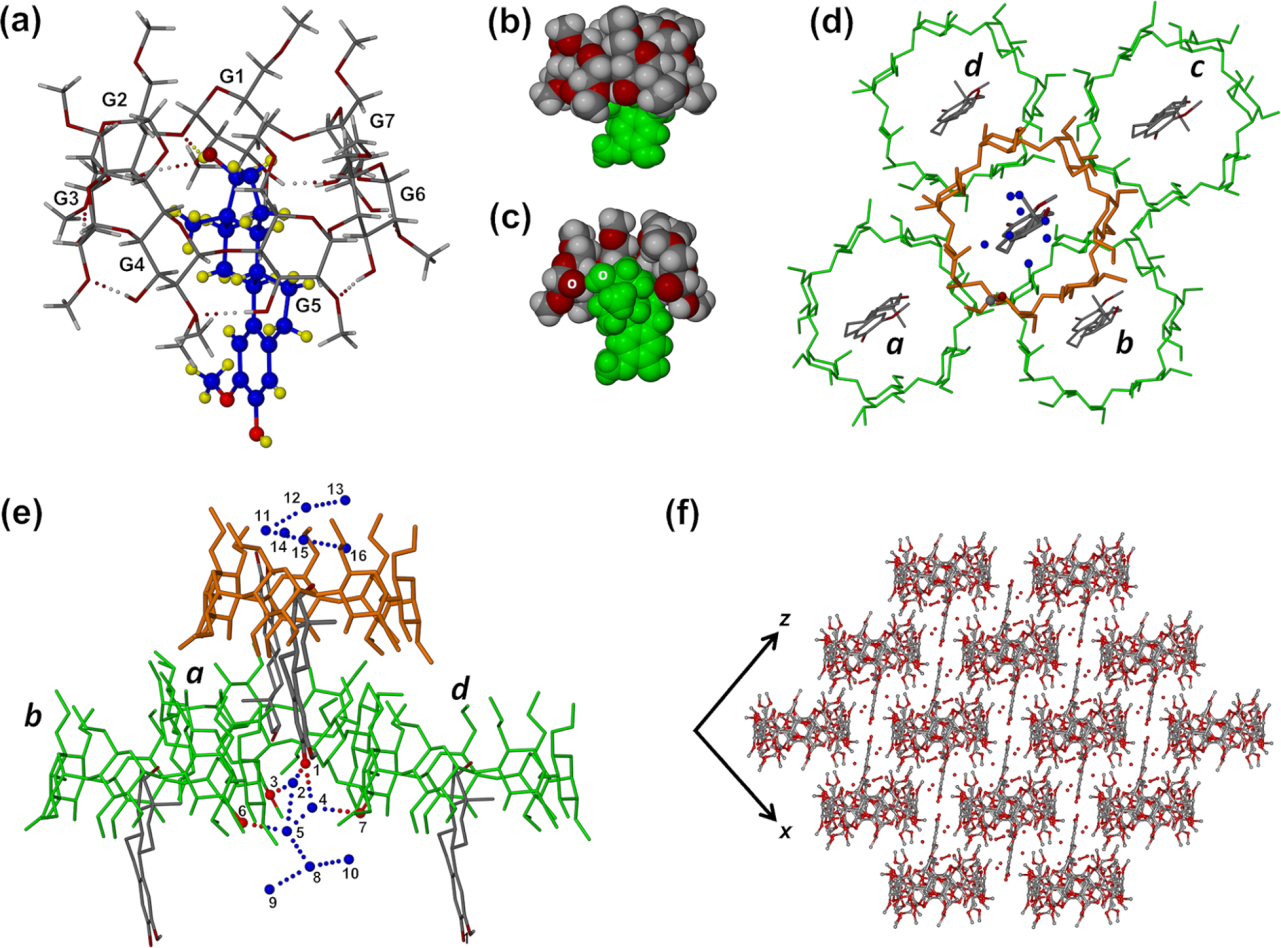
The mode of inclusion of 2ME in the DIMEB cavity (a), space-filling side view of the complex with the 2ME molecule in green (b) and cutaway view (c), location of the A-ring of 2ME protruding from the central DIMEB complex unit (orange) within the space created by the quartet of displaced neighbouring host molecules (d), H-bonding between the representative A-ring hydroxy group –O22–H and three neighbouring CD units mediated by water molecules (blue spheres) (e), and the extended packing arrangement viewed along [10] (f).

Salient features of the mode of inclusion of the steroid molecule 2ME in the host DIMEB molecule and complex crystal packing are shown in Figure 2. In the complex, the DIMEB molecule maintains the ‘round’ conformation that is generally observed for this CD, this feature being effected by the ‘belt’ of intramolecular O2(*n*)–H···O3(*n−*1) hydrogen bonds [23] that link contiguous glucose rings (Figure 2a) and for which the seven relevant O···O distances span the narrow range 2.771(5)–2.883(6) Å (see Table S1 in Supporting Information File 1). The set of seven glycosidic oxygen atoms comprising the so-called ‘O4-heptagon’ has a high degree of planarity, with a root-mean-square deviation (RMSD) from the least-squares plane of only 0.06 Å. Guest inclusion involves entry of the D-ring terminus of the 2ME molecule from the wider (secondary) side of the DIMEB molecule and location of this ring near the narrower (primary) side. Maximal penetration of the D-ring into the CD cavity places atom O19 of the 17-OH group at a distance of ≈0.7 Å above the mean plane of the O4-heptagon, where it engages in a hydrogen bond with one of the host glycosidic oxygen atoms (O19–H···O4G2, with O···O 3.195(6) Å and angle O–H···O 151°). This interaction is complemented by a weaker C–H···O19 hydrogen bond (not shown) involving a host methine group donor (C5G2–H···O19, with C···O 3.280(7) Å and angle C–H···O 143°). A side-view of the complex unit (Figure 2b) in space-filling representation reveals that essentially only the A-ring of the 2ME molecule protrudes significantly from the host secondary side, while the sectioned view (Figure 2c) indicates that the steroid molecule appears to be ‘wedged’ into the CD cavity to its maximal extent. The mean plane of the O4-heptagon and that of the 2ME molecule intersect at ≈84°, this nearly perpendicular relationship being expected under the constraint of the belt of intramolecular hydrogen bonds on the secondary rim of the DIMEB molecule that limits its conformational flexibility, which in turn restricts the orientation of the steroid molecule within the host cavity.

Quantitative indications that the host molecule maintains a fairly symmetrical shape while accommodating the 2ME molecule include the narrow ranges in parameters that measure distortion in CD molecules [24]. These include the radii (*r*) of the O4-heptagon, i.e., the distances from the O4-heptagon centroid to each of the O4 atoms (range 5.02–5.12 Å), the glycosidic O4(*n*)···O4(*n*+1) distances, *D* (range 4.36–4.42 Å), and the O4(*n*−1)···O4(*n*)···O4(*n*+1) angles (*a*) (range 126.6–129.6°). On the other hand, the ‘tilt angle’ parameter (*τ_2_*), measured as the angle between the plane containing atoms O4(*n*), C4(*n*), C1(*n*) and O4(*n*+1) of a given ring and the O4-heptagon mean plane, shows some variation, albeit narrow (range 8.8–14.4°), as the host accommodates the steroid molecule. (A full listing of these and other parameters is provided in Supporting Information File 1). To assess the extent of possible host molecule distortion that the inclusion of a molecule of 2ME in DIMEB might produce, the above tilt angle range for the seven glucose residues in the complex was compared with the ranges calculated for crystalline forms of DIMEB containing either no guest or water molecules only [16]. Interestingly, these ranges span a significantly wider range than that quoted above, namely −5.1–34.6° for anhydrous DIMEB (CSD refcode ZULQAY), 3.6–24.3° for DIMEB dihydrate (CEQCUW) and 2.8–27.6° for DIMEB pentadecahydrate (CSD refcode BOYFOK04), confirming that complexation between 2ME and DIMEB does not cause untoward host distortion, but is instead associated with a more symmetrical macrocyclic shape.

On the the primary side of the DIMEB molecule, three of the torsion angles O5–C5–C6–O6 (*ω*) are (+)-*gauche* and four are (−)-*gauche*, while six of the C5–C6–O6–C9 torsion angles have a *trans* orientation, the exception being that for glucose G5, where the orientation is (+)-*gauche*. This combination results in the methoxymethyl chains on the primary side of the DIMEB molecule being significantly extended and thus providing space directly above the included 2ME molecule (Figure 2d and Figure 2e) that accommodates six components of disordered water molecules. The remaining four components reside outside the DIMEB cavity, where they engage in hydrogen bonding with oxygen atoms on the periphery of the host molecule.

Comparison of the conformation of the encapsulated 2ME molecule and that of the same molecule in its own crystal structure [9] shows that there is minimal difference, with a RMSD of only 0.084 Å and a maximum deviation between two equivalent atoms of 0.202 Å. (A graphical overlay of the molecules appears in the Supporting Information File 1). Thus, in the DIMEB inclusion complex, the steroid skeleton retains the same conformation as that recently observed in the crystal of 2ME, as well as in crystals of the solvated phase 2ME·(CHCl_3_)_2_ and the sulfamoylated derivative of 2ME, namely 2-methoxyestradiol-3,17-di,*O*-bis-sulfamate [9], the A-ring being planar (aromatic), the B-, C- and D-rings being a half-chair, a chair and a twist form (twisted on C13–C14) respectively. Thus, no significant distortions of the host or guest conformations are evident as a result of inclusion of the 2ME molecule in the host DIMEB.

Protrusion of the A-ring of the 2ME molecule from the secondary side of the DIMEB host molecule leads to a unique crystal packing arrangement, details of which are shown in Figure 2d–f. The A-ring of the 2ME molecule in the central complex unit (Figure 2d) is located in an interstitial pocket at the juncture of four neighbouring complex units *a*–*d* in a layer below. As shown in Figure 2e, the A-ring phenolic group –O22–H is hydrogen bonded to a network of water oxygen atoms (blue spheres), three of which act as bridges to the three neighbouring CD molecules (see Table S2 in Supporting Information File 1, for the atom label key, all relevant O···O distances and symmetry operators). In summary, the phenolic group oxygen atom (labelled 1) is hydrogen bonded to water oxygens 2 and 4, which respectively hydrogen bond to O6G2 (3) of host molecule *a* and O3G7 (7) of host molecule *d*. Finally, the water cluster is hydrogen bonded to atom O3G4 (6) of host molecule *b* via water oxygen atom O2 (5). The A-ring is thus strongly tethered within the interstitial pocket. From Figure 2e it is also evident that the water clusters are located in the regions near the two hydrophilic terminals of the steroid molecule (namely the phenolic groups –O22–H on the A-ring and –O19–H on the D-ring). Each 2ME molecule is thus isolated from other 2ME molecules primarily by encapsulation within the DIMEB cavity, but also by hydrogen bonded water clusters that ‘cap’ the terminal rings. The unique layering that results in the extended crystal structure is shown in Figure 2f, a view along the [010] direction.

Methylation of the DIMEB molecule at the 3-position results in fully methylated β-CD (TRIMEB), the second crystalline derivatised CD investigated here for its potential to include the steroid 2ME. Permethylation of DIMEB has the drastic effect of eliminating the belt of O2(*n*)–H···O3(*n−1*) hydrogen bonds observed in that host molecule, so that the conformational constraints described above no longer apply to the resulting host TRIMEB. This indicates the distinct possibility of differences arising in the modes of inclusion of 2ME in TRIMEB relative to that observed in DIMEB, and this was indeed confirmed, as detailed below.

**Figure 3:**
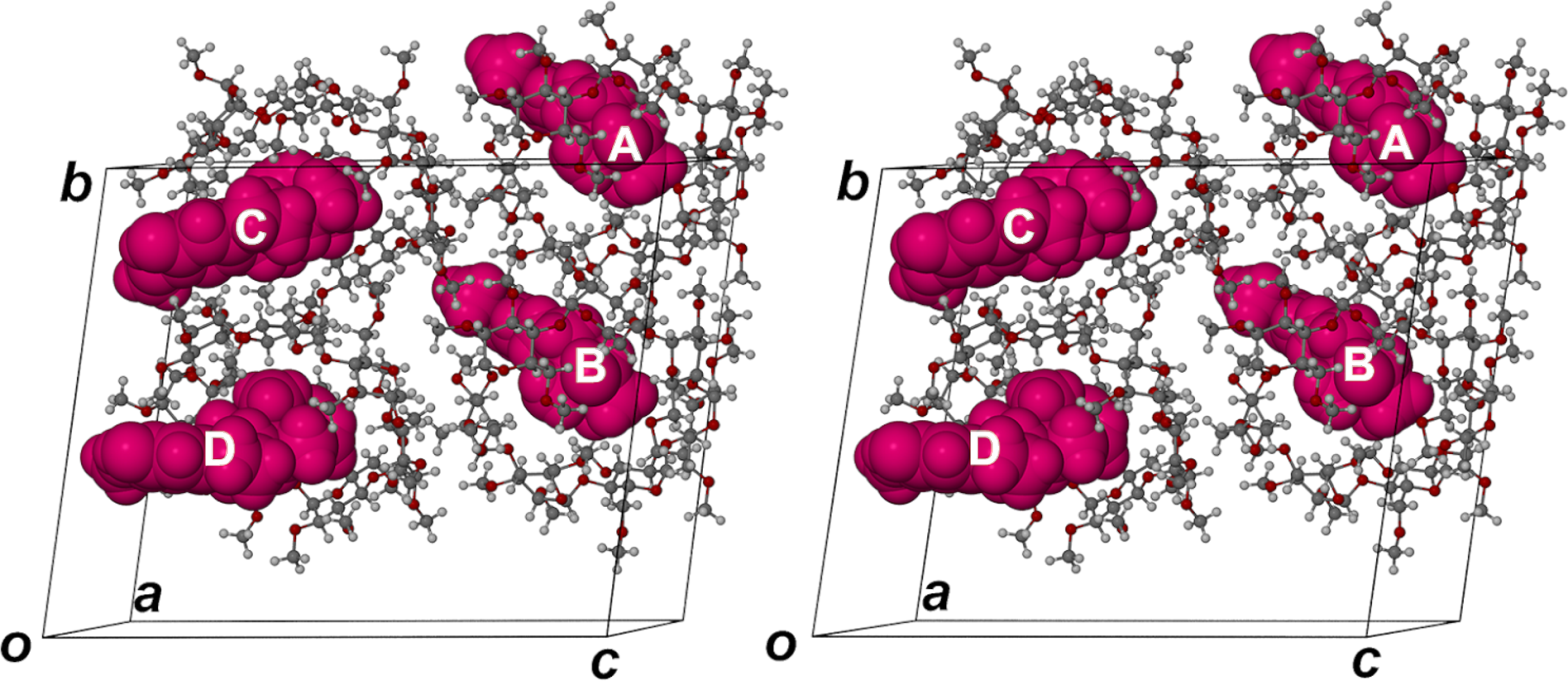
Stereoview of the asymmetric unit in the crystal of the inclusion complex TRIMEB•2ME, with the host TRIMEB molecules drawn in ball-and-stick representation and the guest 2ME molecules (magenta) in space-filling mode.

Whereas the crystal asymmetric unit of the DIMEB complex of 2ME comprises only a single (hydrated) DIMEB·2ME complex unit, the asymmetric unit in the crystal of the inclusion complex between TRIMEB and 2ME (TRIMEB·2ME), consists of four distinct 1:1 host–guest units, shown in the stereoview of Figure 3. Since the space group is *P*1, the asymmetric unit corresponds to the full unit cell content, implying also that each of the complex units A–D is unique, being unrelated to any other by crystallographic symmetry elements.

What is immediately evident from Figure 3 is that the gross features of the mode of 2ME inclusion appear to be essentially the same in all four complex units, the guest molecule entering the secondary side of the TRIMEB molecule, with the D-ring penetrating the cavity maximally, and with both the A-ring as well as a portion of the B-ring protruding from the host secondary side. Further examination of the arrangement of the complex units in Figure 3, however, indicates that the units A and B are very similar in structure and that they are spatially related by a translation of ≈*b*/2, the same translational relationship applying to the pair of complex units C and D. This feature of a ‘pseudo-cell’ having one or more lattice dimensions close to one-half of the true periodicity is common, and is often induced when a crystal initially at ambient temperature is cooled for X-ray data-collection. In the case of the TRIMEB·2ME complex, however, the unit cell dimensions of the crystal determined initially at 21 °C corresponded with those recorded at −100 °C, the temperature of the data-collection. During attempts to solve the structure, the presence of such a ‘pseudo-cell’ for the TRIMEB·2ME crystal was predicted from a preliminary examination of the X-ray diffraction pattern, which showed that the reciprocal lattice layers *hkl* with *k* = *2n* had significantly higher intensities than those with *k* = *2n + 1* (see Supporting Information File 1 for images of representative reciprocal lattice levels). Within the above temperature range, therefore, there are discernible structural differences among the four TRIMEB·2ME complex units, as detailed below, such differences being of interest in the context of ‘mutually induced fitting’ of host and guest during complexation [1].

**Figure 4:**
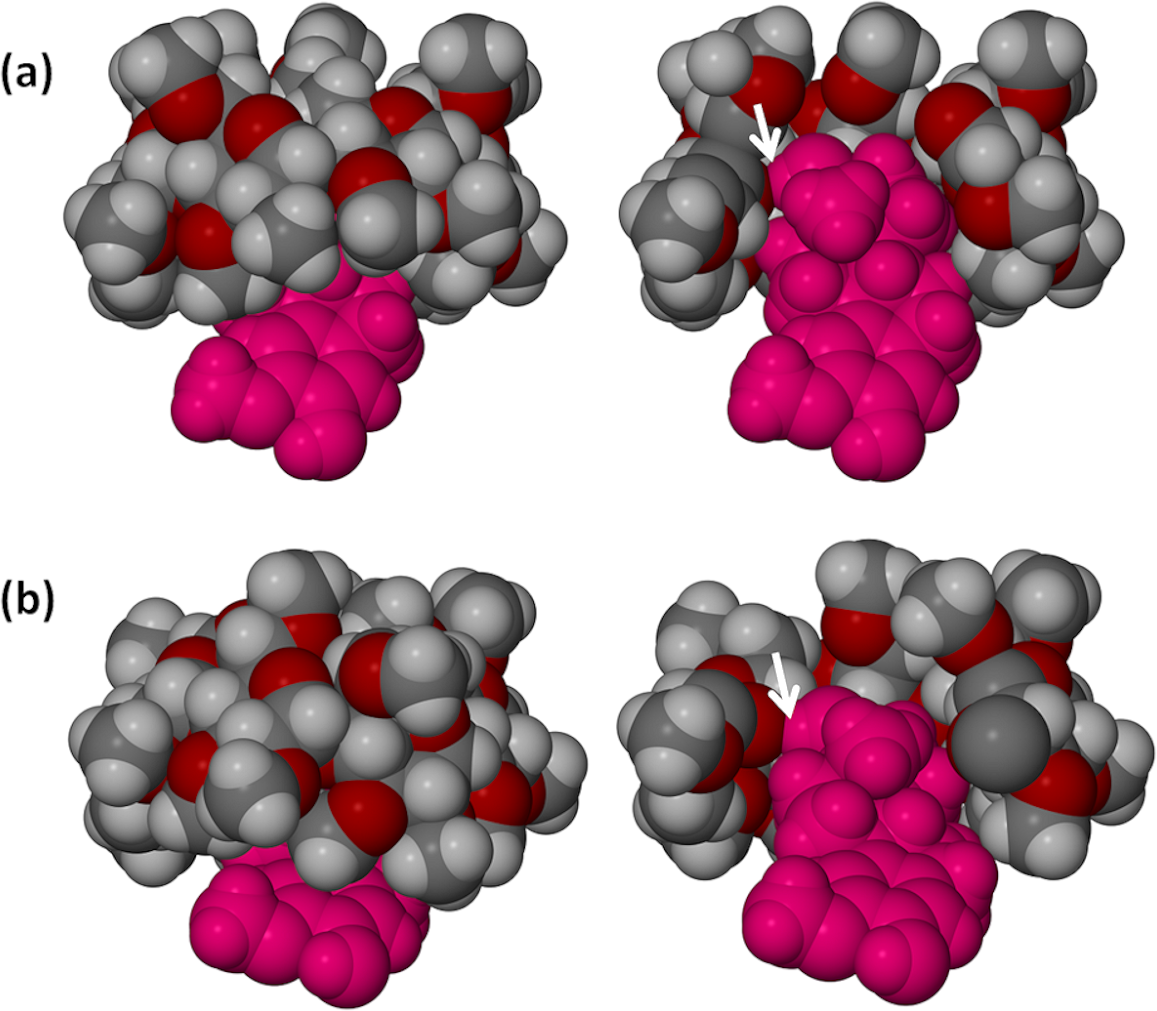
Space-filling images of complex unit A (as representative of units A and B) (a) and complex unit C (b) of TRIMEB·2ME. The images on the left show guest residues (magenta) protruding from the secondary side of the host molecule while the images on the right are cutaway views highlighting the common (guest)O19–H···O4(host) hydrogen bond, indicated by arrows.

Complex units A and B of TRIMEB·2ME, related by a pseudo-translation of ≈*b*/2, have nearly identical structural features, namely common TRIMEB host conformations and lack of host disorder, apparent twofold disorder of the 2ME methylene group at C6 (site-occupancy factors, s.o.f.s, 0.56 and 0.63 for the respective major components) and essentially the same mode of guest inclusion. (A full listing of these and other parameters is provided in Supporting Information File 1). A least-squares overlay of the host molecules A and B (see Supporting Information, File 1) yields a RMSD of only 0.157 Å and a maximum deviation between two equivalent atoms of 0.649 Å. In both cases, the phenolic group –O19–H of 2ME is hydrogen bonded to a common glycosidic oxygen atom, with O···O distances and O–H···O angles having values of 3.064(6) Å and 169° respectively for complex unit A, and 3.186(6) Å and 170° for unit B. Figure 4a, featuring complex unit A as representative, illustrates in space-filling mode the nature of the common guest inclusion mode in complex units A and B. This involves encapsulation of the C- and D-rings of 2ME within the host cavity while the A- and B-rings protrude from the secondary side of the TRIMEB molecule. The hydrogen bond that links the host and guest molecules is indicated in the cutaway view of Figure 4a.

In contrast to the symmetrical host in the DIMEB·2ME complex, where the –C5–CH_2_–O–CH_3_ residues on the primary side of the host are fully extended and the tilt angles of the glucose rings are not severe, the strong tilting of the glucose rings of the TRIMEB·2ME complex results in several of these residues being in close contact. This results in steric crowding of the primary methoxy groups, creating a bowl-like surface on the host primary side above the D-ring of the included 2ME molecule. Such a bowl-like surface was first observed for the host DIMEB [25] and was subsequently observed in numerous inclusion complexes of TRIMEB as well [16].

The host molecules in complex units C and D (also displaced by ≈*b*/2) are nearly isostructural, but differ significantly from the isostructural pair A and B, as indicated by the conformational parameters described earlier. The least-squares overlay of host molecules C and D reveals several differences in the torsional parameters of the primary methoxy residues (see Figure S15, Supporting Information File 1), which result in a RMSD of 0.819 Å and a maximum deviation of 3.417 Å between two terminal methoxy carbon atoms. Figure 4b illustrates in space-filling mode the inclusion of 2ME in host molecule C as representative. Guest inclusion again involves hydrogen bonding between the phenolic group O19–H and a host glycosidic O4 atom (O···O 2.894(6) Å). This is evident in the cutaway diagram in Figure 4b.

Complex unit C is unique in having neither host nor guest disorder and complex unit D also shows a unique feature, namely disorder in both the C- and D-rings of the 2ME molecule, which is thus present as two conformers in the ratio 0.61:0.39. One consequence of this is that there are two distinct positions for the O19–H phenolic group, each conformer forming a hydrogen bond with a different TRIMEB acceptor oxygen atom. Further details of this disorder and its implications are given below.

**Figure 5:**
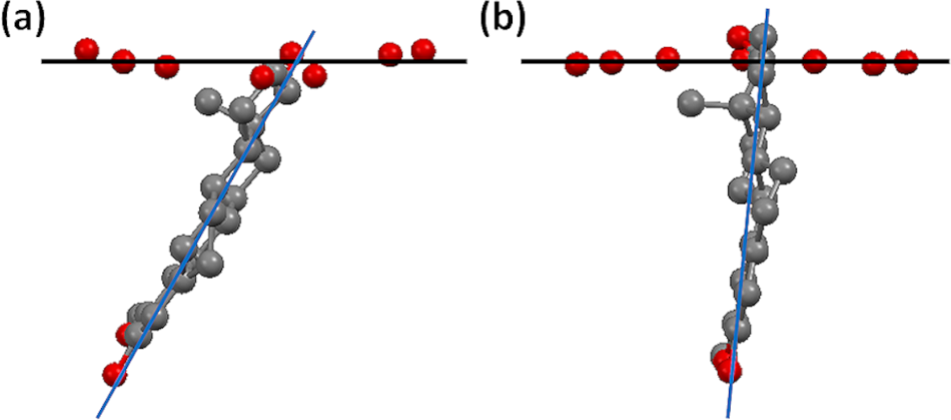
Inclination of the mean plane of the included 2ME molecule relative to the O4-heptagon of the host molecule in (a) complex unit A of TRIMEB·2ME, (b) the complex DIMEB·2ME. The respective interplanar angles are ≈60° and ≈84°.

Another very significant feature is evident from comparing the structures of the TRIMEB·2ME and DIMEB·2ME complexes, namely the inclination of the mean plane of the included steroidal guest molecule relative to the mean O4-plane of the respective host molecule as reference (Figure 5). The interplanar angles listed in the caption contrast the relatively ‘shallow’ entry of the 2ME molecule into the secondary side of hosts A and B of TRIMEB with its nearly ‘vertical’ entry into the host DIMEB. Furthermore, the corresponding parameters for inclusion of 2ME in the TRIMEB hosts C and D are in the narrow range ≈45–49°, i.e., significantly different from the ≈60–61° observed for inclusion in TRIMEB host molecules A and B. Thus, inclusion of the 2ME molecule into the secondary sides of hosts C and D is even more ‘shallow’ than it is for the hosts A and B, consistent with the finding that these pairs of host molecules have significantly different conformations.

**Figure 6:**
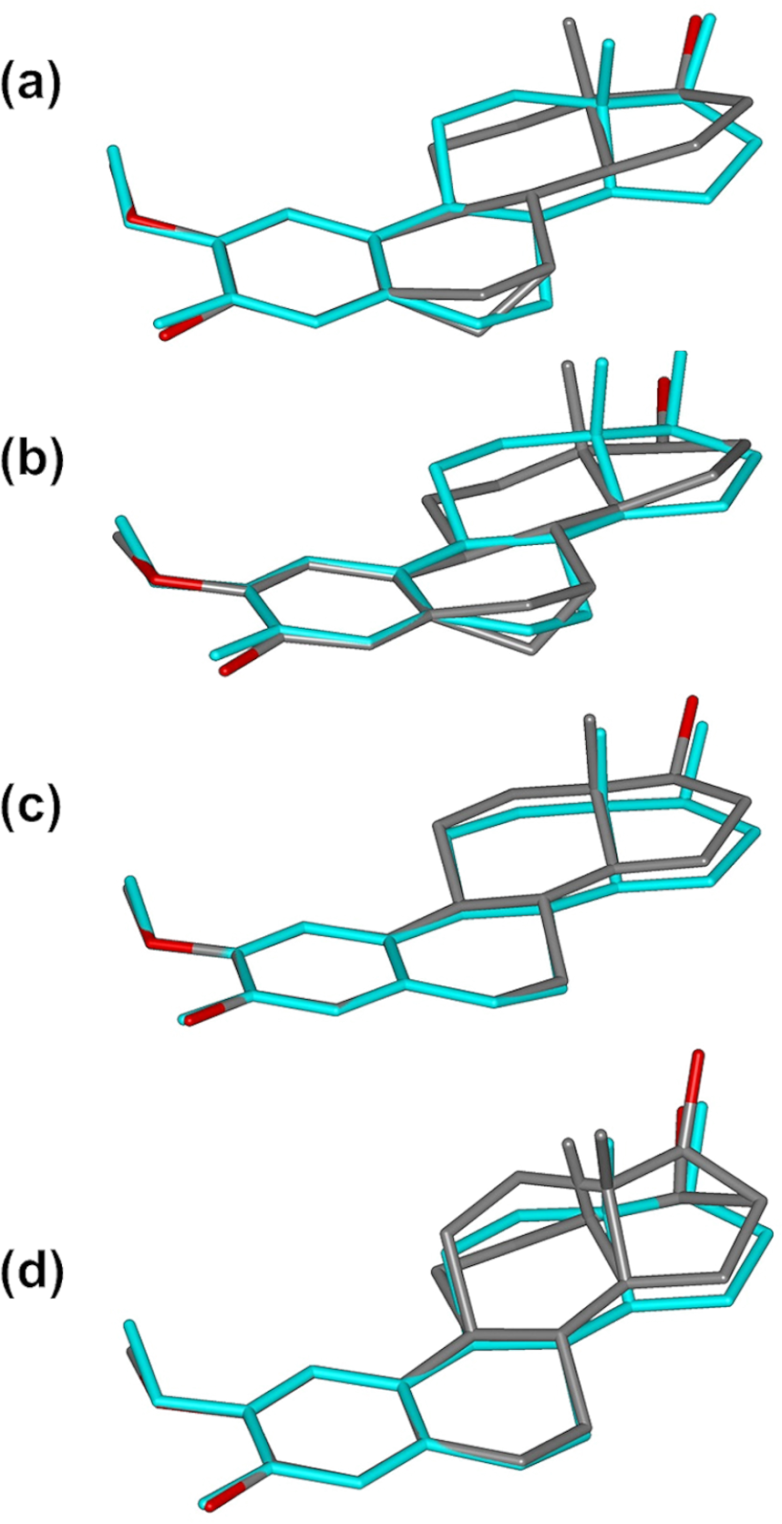
Overlay of the steroid nucleus of 2ME (blue) in its own crystal with the refined models (grey) of the four molecules of 2ME in the complex units A (a), B (b), C (c), D (d) of TRIMEB·2ME.

The steroid molecules included in the respective complex units A–D are shown in Figure 6 where each is overlayed on the steroid nucleus taken from the crystal structure of 2ME alone [9], the comparison being based on maximum overlap of the A-ring (aromatic) atoms in each case. In the 2ME molecule itself, as the common reference, the B–D ring conformations are a half-chair (B-ring), a chair (C-ring) and a twist-form, twisted on C13–C14 (D-ring) [9]. The refined guest models in Figure 6a and Figure 6b are virtually identical, mirroring the close similarity of the conformations of their respective host molecules in complex units A and B. The methylene carbon atom C6 on ring B showed abnormally high thermal motion which could not be modelled satisfactorily with anisotropic displacement parameters; it was therefore treated as a ‘split’ atom, resulting in s.o.f. ratios of 0.44:0.56 and 0.37:0.63 for the disordered components of the C6-methylene group in 2ME molecules A and B. (In these cases, if the two discrete positions indicated for C6 were to be considered for this group, the B-ring conformations would resemble a chair and a twist-boat form, rather than a half-chair).

Ring C adopts a chair conformation (Figure 6a and Figure 6b), while the D-ring is a twist form (twisted on C13-C14). The net result is that the modelled 2ME molecules in complex units A and B have a conformation which is significantly distorted relative to that of the 2ME molecule in its own crystal.

As shown in Figure 6c, the modelled 2ME molecule in complex unit C of the TRIMEB·2ME structure is unique in displaying no evidence of disorder, with the conformations of the B-, C- and D-rings being a half-chair, a chair and a twist form (twist on C13–C14) respectively. While the individual ring conformations correspond with those of 2ME in its own crystal, the overall conformations are clearly not identical, but very close. The conformation of the refined 2ME model in Figure 6c is also very close to that of the 2ME molecule in the DIMEB·2ME complex, with a least-squares fit having a RMSD vaue of 0.083 Å and a maximum atomic deviation of 0.161 Å (see Supporting Information File 1 for graphical overlay).

**Figure 7:**
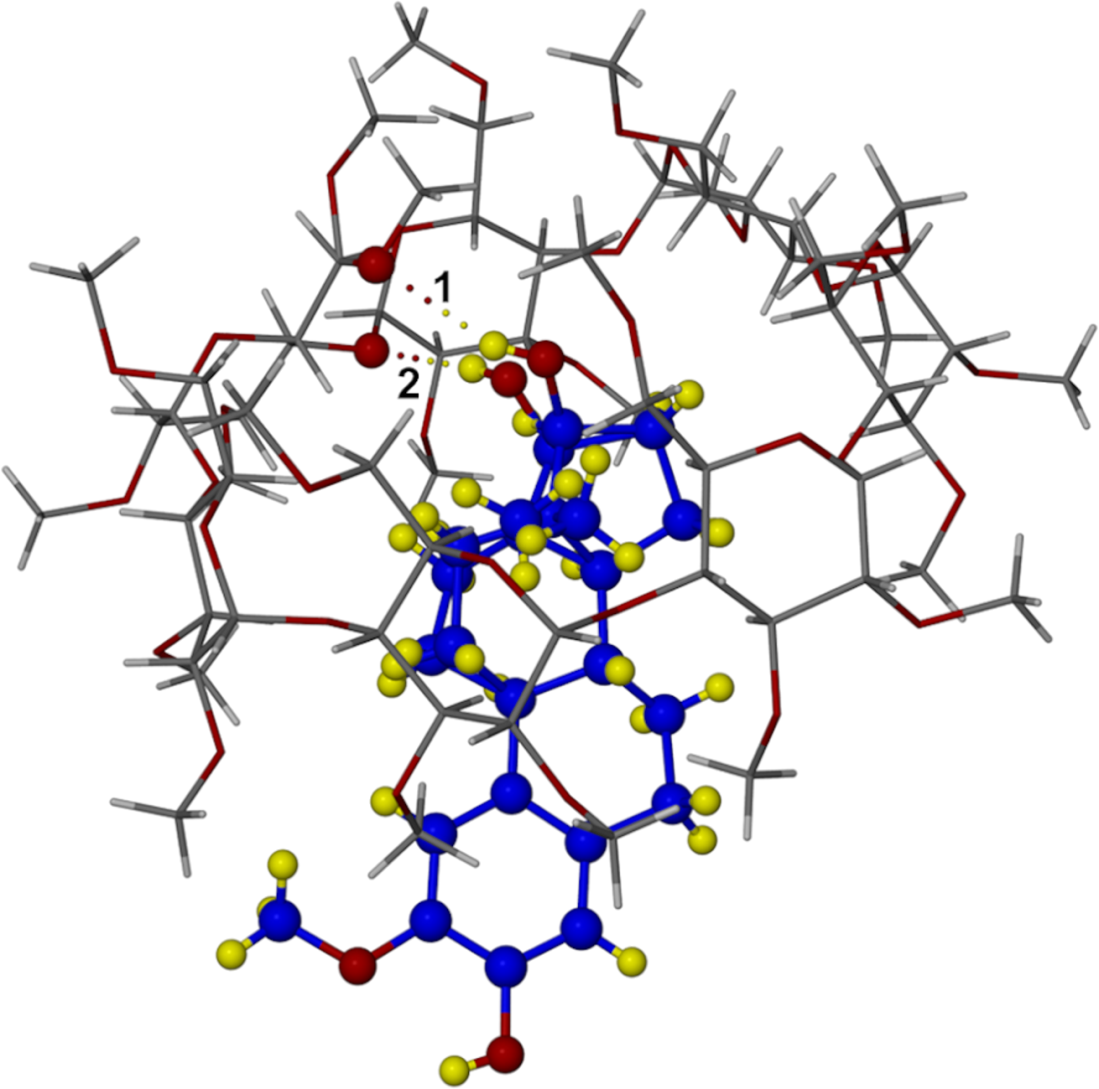
The TRIMEB·2ME complex unit D with two distinct (guest)O19-H···O(host) hydrogen bonds highlighted. To distinguish the host and guest, C and H atoms of 2ME are coloured blue and yellow respectively.

It was noted above that the least-squares overlay of host molecules A and B yielded a considerably smaller RMSD (0.157 Å) than the overlay of host molecules C and D (0.819 Å). Accordingly, the conformation of the modelled 2ME molecule in complex unit D differs from that in complex unit C. Specifically, in complex unit D (Figure 6d) the steroid molecule displays significant disorder of both the C- and D-rings, the C-ring adopting as major component the chair form (s.o.f. 0.61) and as minor component a slightly twisted chair (s.o.f. 0.39), while the D-ring adopts as major component a twist-form (twisted on C13D–C14D) and as minor component an envelope (flap on C17F) with respective s.o.f.s as for ring C. An interesting consequence of the presence of two guest conformers in complex unit D is that the disordered components of the 17-OH group are separated by ≈1.5 Å, one as the major component (s.o.f. 0.61) and the other as the minor, with s.o.f. 0.39 and these –OH groups are donors in different guest–host hydrogen bonds. This feature is illustrated in Figure 7, where the respective H-bonds indicated are as follows: **1** – (major component O19D)–H···O6D5 with O···O 3.07(1) Å and O–H···O angle 152°; **2** – (minor component O19F)-H···O4D5 with O···O 2.96(2) Å and O-H···O angle 172°.

The structures of the hydrated DIMEB·2ME and the TRIMEB·2ME complexes presented here illustrate for the first time details of the encapsulation of a ‘classical’ steroidal molecule by cyclodextrin hosts. In addition, from the variety of host and guest conformations observed in the TRIMEB·2ME complex, even in one crystal, it is evident that complex formation involves some degree of ‘mutually induced fit’ of host and guest [1,26].

### Enhancement of the solubility of 2ME by selected CDs

One of the objectives of our study of the anticancer agent 2ME was to investigate methods of improving its aqueous solubility (*S*_o_ = 0.005 mg/cm^3^ at 25 °C [9]) which might in turn enhance its poor bioavailability [4–5]. A factor which limited investigation to the relatively simple initial analysis reported here was the prohibitive cost of synthesis of 2ME and hence the paucity of material available. This precluded our carrying out, e.g., full phase-solubility analyses, which would have yielded additional information such as the values of the association constants for complex formation in solution. Thus instead, the simpler expedient of determining the solubility of 2ME in a series of aqueous solutions of both native and derivatised cyclodextrins of known concentrations (typically 2% m/v) was initially employed in order to estimate solubility enhancement factors (*S*_CD_/*S*_o_, where *S*_CD_ is the solubility of 2ME in the CD solution at the same temperature). This parameter has been used previously to gauge the ability of a range of CDs to solubilise drugs with poor aqueous solubility [27]. The cyclodextrins employed and the resulting apparent solubilities relative to that of 2ME in water are summarised in Table 2.

**Table 2:** Solubility enhancement factors^a^ for 2ME with various cyclodextrins at 25 °C.

cyclodextrin	CD solution concentration (% *m*/*v*)	solubility enhancement factor *S*_CD_/*S*_o_

α-CD	2	7.5
β-CD	2	10
γ-CD	2	86
RAMEB (randomly methylated β-CD)	2	156
DIMEB (heptakis(2,6-di-*O*-methyl)-β-CD)	2	256
TRIMEB ( heptakis(2,3,6-tri-*O*-methyl)-β-CD)	2	23
HPBCD (hydroxypropyl-β-CD)	2	88
TRIMEA (hexakis(2,3,6-tri-*O*-methyl)-α-CD)	0.5	4
TriAcBCD (triacetyl-β-CD)	0.002	8
TriAcGCD (triacetyl-γ-CD)	0.0025	2
TriEtGCD (triethyl-γ-CD)	0.002	0.84

^a^All experimental data were compared with those for a control and were based on triplicate measurements.

Interestingly, with the exception of TriEtBCD, all of the CDs tested yielded some improvement in 2ME solubility. Of the native CDs, γ-CD was the most efficient solubiliser for 2ME, a result which is consistent with earlier studies for a variety of steroidal guests [28]. However, the best enhancement was achieved with the derivatised CDs DIMEB, RAMEB, HPBCD and TRIMEB. As γ-CD, RAMEB and HPBCD are more pharmaceutically acceptable [29], they therefore appear to be media with potential use in medicinal applications requiring elevated concentrations of 2ME.

**Figure 8:**
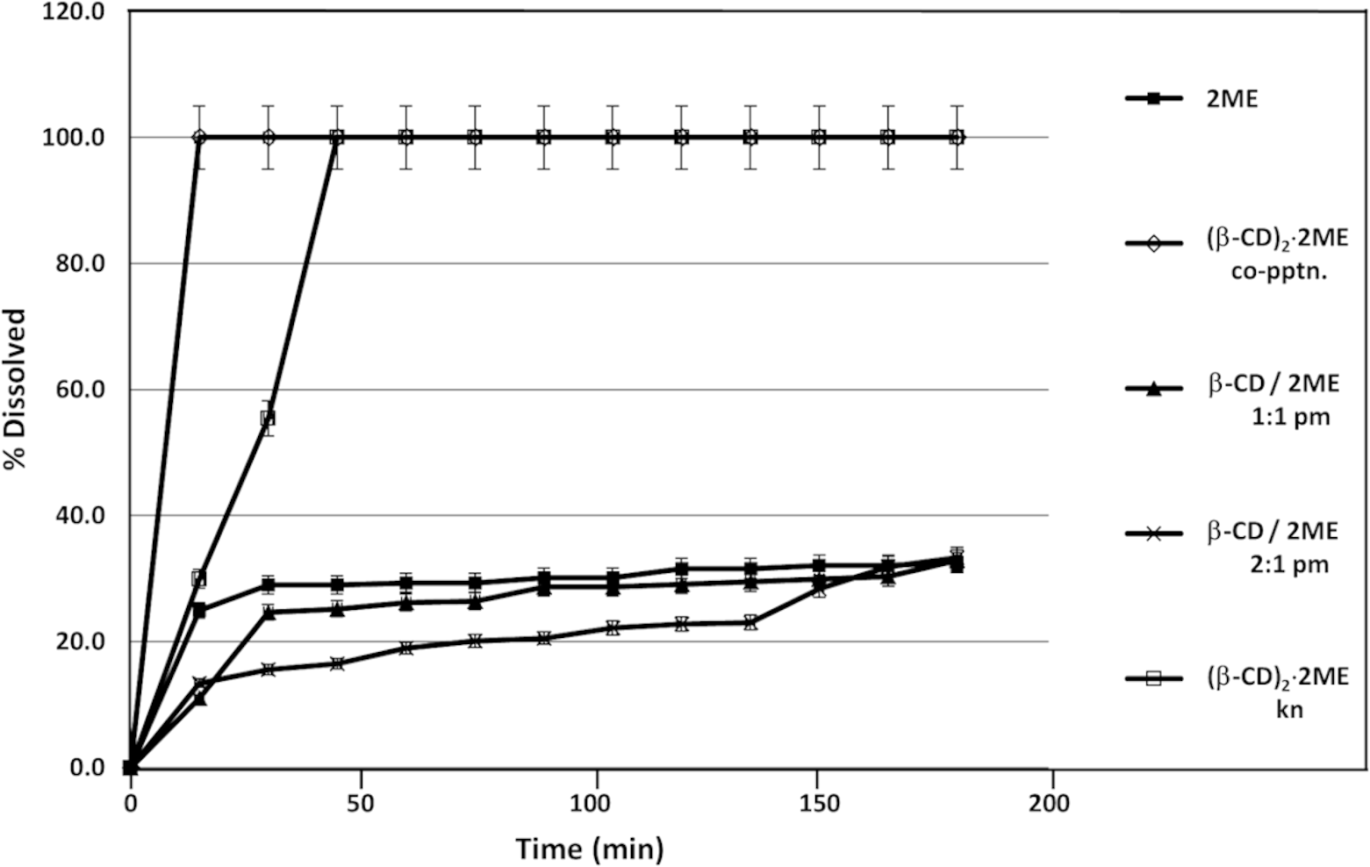
Dissolution profiles in water at 37 °C for untreated 2ME and various β-CD-containing preparations of 2ME.

Comparative dissolution rate studies at 37 °C using the rotating basket method with samples in gelatin capsules were also carried out for pure 2ME and various preparations containing β-CD and 2ME (Figure 8), namely the hydrated 2:1 inclusion complex obtained by co-precipitation, two physical mixtures (pm) of β-CD and 2ME (molar ratios 1:1 and 2:1), and the hydrated 2:1 inclusion complex obtained by kneading (kn). Of all the preparations tested, 2ME in the form of its β-CD inclusion complex, prepared by both co-precipitation and kneading, yielded the most rapid and complete dissolution, the times for attaining the 100% level being 15 and 45 minutes, respectively. This performance significantly outweighed that of the untreated 2ME, for which the percentage dissolved attained a maximum level of only 25–32% after 15 minutes. The dissolution of 2ME from physical mixtures of β-CD and 2ME was evidently hindered by the presence of the cyclodextrin, an effect that increased with the concentration of β-CD present.

Comparative dissolution profiles were also recorded for 2ME in physical mixtures with the native cyclodextrins β- and γ-CD as well as RAMEB and HPBCD (Supporting Information File 1). While only 30% of untreated 2ME dissolved in water after 20 minutes, the equimolar γ-CD/2ME physical mixture yielded a value of ≈55% during the same period. Furthermore, after three hours, the level had risen to 80%. In contrast, the other CD/2ME mixtures produced relatively small effects on the release of 2ME. It was concluded that the γ-CD/2ME physical mixture had potential as a vehicle for formulation of a controlled release form of 2ME.

Finally, it was considered of interest to compare the dissolution profile of the inclusion complex (β-CD)_2_·2ME (shown in Figure 8 to be very favourable) with those for a series of 2ME/CD samples resulting from kneading (kn) (Supporting Information File 1). This series included the products of kneading in 1:1 molar ratios each of the combinations 2ME with α-CD, with β-CD and with γ-CD, as well as the 1:1 inclusion complexes HPBCD·2ME and RAMEB·2ME. All of these preparations led to rapid dissolution of 2ME. In particular, both the γ-CD/2ME and the RAMEB/2ME samples resulted in 100% of the 2ME concentration being attained 15 min after the commencement of the dissolution run, as found also for the complex (β-CD)_2_·2ME. All of these preparations displayed superior dissolution characteristics to those of untreated 2ME, only 30% of which dissolved in the same medium within 20 minutes.

## Conclusion

Single crystal X-ray diffraction has revealed the modes of inclusion of the potent anticancer agent 2-methoxyestradiol (2ME) in two representative host CD molecules, one of them (DIMEB) having restricted conformational flexibility owing to cooperative intramolecular O–H···O hydrogen bonding that maintains the round shape of the macrocycle, and the other (TRIMEB) lacking this facility and hence having access to significantly greater conformational freedom. Accordingly, in the case of the complex DIMEB·2ME, while the host molecule is relatively undistorted on inclusion of 2ME, the guest molecule maintains the conformation that it displays in its own crystal structure. Instead, in the case of the TRIMEB·2ME complex, the four independent 1:1 host–guest units in the crystal display a variety of host distortions as well as guest conformational variation, reflecting a mutually induced fit accompanying complexation. The phenomenon of mutually induced fit was highlighted previously by Mavridis et al. for per-derivatised CDs and especially in the case of TRIMEB [30]. The TRIMEB·2ME complex is thus another example of a synthetic system that displays host–guest adaptation on complexation, complementing our recently reported analogous observations for inclusion complexes between methylated CDs and the antioxidant *trans*-resveratrol [26]. Hydrogen bonding between the 17-OH group of 2ME and glycosidic (O4) oxygen atoms within the cavity of the host CDs generally occurs, while in unit D of the TRIMEB complex, where there are two 17-OH disorder components, the major component hydrogen bonds to a host methoxy O6 atom and the minor to a host glycosidic oxygen atom. The results presented here are of general interest in the context of CD–steroid interaction, with special reference to estranes that contain a 17-OH function (e.g., the estrogen sex hormone 17β-estradiol).

Previous studies of the anticancer activities of 2ME have indicated that its low aqueous solubility and rapid metabolism lead to poor bioavailability [4–5]. However, the effect of various CDs on the aqueous solubility of 2ME has hitherto not been assessed. In this study, we have shown via a variety of in vitro experiments that CDs can enhance 2ME aqueous solubility very significantly and conclude that they warrant consideration as potential vehicles for facilitating both further biological testing of 2ME in anticancer studies as well as the development of alternative dosage forms of 2ME.

## Experimental

### Materials

2-Methoxyestradiol (2ME, C_19_H_26_O_3_) was generously provided by Shimoda Biotech (Pty) Ltd (Port Elizabeth, South Africa). Synthesis of the sample of 2ME from commercially available 17β-estradiol was reported earlier [9]. The following cyclodextrins (CDs) were purchased from Cyclolab (Budapest, Hungary) and were used as received: α-CD, β-CD, γ-CD, hexakis(2,3,6-tri-*O*-methyl)-α-CD, heptakis-(2,6-di-*O*-methyl)-β-CD, heptakis(2,3,6-tri-*O*-methyl)-β-CD, randomly methylated β-CD, heptakis(2,3,6-tri-*O*-ethyl)-β-CD, heptakis(2,3,6-tri-*O*-acetyl)-β-CD and octakis(2,3,6-tri-*O*-acetyl)-γ-CD. All other materials and solvents used were of analytical reagent grade.

### Complex preparation

The inclusion complex (β-CD)_2_·2ME·12.6H_2_O was generated within 45–60 min via the manual kneading technique with the host and guest in a 2:1 molar ratio and using only water as liquid medium. The same crystalline phase was obtained by co-precipitation, which involved vigorous stirring at 60 °C of an aqueous solution containing the host and guest in a 2:1 molar ratio. Slow cooling yielded single crystals of the inclusion complex. Complexation between 2ME and the amorphous hosts RAMEB and HPBCD was achieved by kneading (as above) but with a 1:1 host–guest molar ratio.

The crystalline inclusion complex DIMEB·2ME was prepared as follows. A 44.00 mg (0.033 mmol) sample of DIMEB and 10 mg (0.033 mmol) of 2ME were placed in 3 cm^3^ of distilled water and the suspension was stirred vigorously over a period of 24 h at ambient temperature (18–25 °C). The resulting solution was filtered (0.45 μm nylon microfilter) and then placed on a hot plate set at 60 °C. After several days, large block-like single crystals appeared and were harvested for analysis. Identical procedures were successfully employed to isolate single block-like crystals of the TRIMEB inclusion complex of 2ME, TRIMEB·2ME, the only difference being the mass of the host compound TRIMEB (44.27 mg, 0.033 mmol).

### Thermal analysis

HSM was performed on a Linkam TH MS600 hot stage connected to a Linkam TP92 temperature control unit. Images were captured by a real-time Sony Digital Hyper HAD colour video camera coupled to a Nikon SMZ-10 stereoscopic microscope. The Soft Imaging system, analySIS [31] was used to process the images.

For TGA a Mettler Toledo TGA/SDTA851e instrument was employed with a nitrogen gas purge (flow rate 30 cm^3^/min). Indium (mp 156.6 °C) and aluminium (mp 660.3 °C) were used for all calibrations. Accurately weighed samples (mass range 2–5 mg) were placed in an open porcelain pan and were heated at 10 K/min.

For DSC studies, a Perkin-Elmer PC-7 series thermal analysis system was used, with a a nitrogen gas purge (flow rate 30 cm^3^/min). Temperature calibration was achieved using the onset temperatures of fusion of indium (156.6 °C) and zinc (419.5 °C). The heat flow calibration was based on the value of Δ*H* of fusion of indium (28.62 J/g). Samples in the form of fine powders (mass range 2–5 mg) were sealed in crimped, vented aluminium pans and were heated at 10 K/min, with a sealed, empty pan as reference.

### FTIR spectroscopy

A Perkin-Elmer 100 FTIR instrument fitted with UATR and controlled with Spectrum^®^ software for sample analysis was used to record the infrared spectra of crystalline and amorphous powders in the range 400–4000 cm^−1^.

### UV–visible spectrophotometry

Spectra were recorded on a Beckman DU® 640 series UV–vis spectrophotometer operating at a scan rate of 1200 nm/min over the range 190–450 nm with solutions for analysis placed in 2 mm quartz cuvettes. For CD inclusion compounds of 2ME, host–guest stoichiometries were determined at the absorption maximum for the bioactive compound (λ_max_ = 198.5 nm) in methanol/water (1:1 v/v) solution. This instrument was also used for solubility and dissolution rate assays (details below).

### Single crystal X-ray diffraction analysis

Intensity data were collected on a Nonius Kappa CCD diffractometer using graphite-monochromated Mo Kα-radiation (λ = 0.71073 Å), with each crystal coated in Paratone oil (Exxon Chemical Co., TX, USA), mounted on a cryoloop and cooled to 173(2) K in a constant stream of nitrogen vapour (Oxford Cryostream, UK).

Due to the fact that both the DIMEB·2ME and the TRIMEB·2ME inclusion complexes had no previously published isostructural counterparts [16], their structures were solved using ab initio direct methods (program SHELXD [22]) and their refinement by full-matrix least-squares followed with program SHELXH-97 [22] operating under the X-Seed interface [32]. For the DIMEB·2ME structure, with a single complex formula unit DIMEB·2ME in the asymmetric unit, all non-H atoms refined anisotropically except O19 on the D-ring of the 2ME molecule, two disordered methyl groups and several partial water molecules that resulted from the 5.3 water molecules per complex unit being located over ten sites. The former showed excessive thermal motion that was not amenable to standard anisotropic treatment while the disordered water oxygen atoms did not warrant anisotropic treatment. A minor, but unexpected feature of the DIMEB inclusion complex, was partial methylation of the O3-H hydroxy group of the host glucose ring G1 (s.o.f. = 0.5 for the resulting –CH_3_ residue). While this contribution did not lead to any abnormally short intermolecular contacts, such a contact was observed elsewhere, specifically between a disordered methyl group component (s.o.f. 0.5) on residue G6 and a methyl group on glucose residue G7 of a neighbouring host molecule. This necessitated reduction of the s.o.f. of the latter methyl group from 1.0 to 0.5. The net effect was that despite partial methylation of O3–H on G1, appropriate modelling of disordered residues resulted in retention of the nominal formula for the DIMEB molecule, namely C_56_H_98_O_35_. The structure of TRIMEB·2ME, with four distinct complex units in the asymmetric unit, was modelled with anisotropic thermal parameters for all atoms except (a) the carbon atoms of the glucose rings, which refined with relatively low values for their *U_iso_* values (range 0.024–0.053 Å^2^) and showed no evidence of anisotropic motion in difference Fourier syntheses, and (b) ten atoms of the 2ME molecules with partial occupation. Despite the resulting significant reduction in the number of *U_ij_* least-squares parameters, the final structural model was based on full-matrix refinement of as many as 3644 parameters. Hydrogen atoms were sought in difference Fourier syntheses and a large proportion of these could be located. All of the H atoms were included in idealised positions in a riding model with *U_iso_* values in the range 1.2–1.5 times those of their parent atoms. Further details of the data-reduction and least-squares refinements for the complexes appear in the CIF files. CCDC 1428344 and 1428345 contain the supplementary crystallographic data for this paper. These data can be obtained free of charge at http://www.ccdc.cam.ac.uk/products/csd/request/ [or from the Cambridge Crystallographic Data Centre (CCDC), 12 Union Road, Cambridge CB2 1EZ, UK; fax: +44(0)1223-336033; email: deposit@ccdc.cam.ac.uk].

### Solubility and dissolution rate measurements

Aqueous solutions of a series of native and derivatised CDs with pre-determined fixed concentrations were prepared at 25 °C. Following their saturation with 2ME, assaying of the 2ME concentration in the filtered solutions was performed using UV spectrophotometry at 198 nm, with solutions appropriately diluted to ensure that the absorbance values were in the range 0 to 1. A standard calibration curve of absorbance as a function of 2ME concentration was employed. Solubility enhancement factors for the steroid were calculated as *S*_CD_/*S*_o_, where *S*_CD_ is the maximum concentration of 2ME in each CD solution in mg/cm^3^ and *S*_o_ is the aqueous solubility of 2ME in water at 25 °C (viz. 0.005 mg/cm^3^ at 25 °C).

Dissolution studies were carried out using the rotating basket method with samples in gelatin capsules and employing the procedures and instruments we described previously [9]. Recording of dissolution profiles was performed in triplicate. UV spectrophotometric anaysis of 2ME followed the procedure already described above. Analysis of variance (ANOVA) methods were used to discriminate dissolution profiles.

## Supporting Information

File 1 Additional data.
