# Supplementary material for: Inclusion complexes of 2-methoxyestradiol with dimethylated and permethylated β-cyclodextrins: models for cyclodextrin–steroid interaction
Source: Beilstein J Org Chem. 2015 Dec 16;11:2616–30. doi: 10.3762/bjoc.11.281 (PMC4685897; doi:10.3762/bjoc.11.281)
Supplement: File 1 — Additional data. [file Beilstein_J_Org_Chem-11-2616-s001.pdf]

Supporting Information

for

Inclusion complexes of 2-methoxyestradiol with dimethylated and permethylated β-cyclodextrins: models for cyclodextrin–steroid interaction

Mino R. Caira*[,1], Susan A. Bourne[1], Halima Samsodien[1] and Vincent. J. Smith[1]

Address: [1] Centre for Supramolecular Chemistry Research (CSCR), Department of Chemistry, University of Cape Town, Rondebosch 7701, South Africa

Email: Mino R. Caira* - Mino.Caira@uct.ac.za

Additional data

CONTENTS:

1. PXRD traces and single crystal XRD data confirming complex formation between β-CD and 2ME (p. s2)
2. Relevant PXRD traces, DSC traces and FTIR spectra confirming complex formation between the hosts RAMEB and HPBCD and the guest 2ME (p. s3)
3. Thermoanalytical characterization of the DIMEB·2ME and TRIMEB·2ME inclusion complexes (p. s4)
4. Geometrical data for the host DIMEB in the inclusion complex DIMEB·2ME (Table S1) (p. s6)
5. Molecular overlay of 2ME molecules (uncomplexed and complexed with DIMEB) (p. s7)
6. Additional hydrogen bond data for DIMEB·2ME (Table S2) (p.s s8)
7. Simulated X-ray photographs for the TRIMEB·2ME complex (p. s9)
8. Overlay of host molecules A and B in the TRIMEB·2ME complex (p. s9)
9. Overlay of host molecules C and D in the TRIMEB·2ME complex (p. s10)
10. Geometrical parameters for the host molecules A–D in the inclusion complex TRIMEB·2ME (Table S3) (p. s10)
11. Overlay of the 2ME molecules in TRIMEB·2ME complex unit C and the DIMEB·2ME complex (p. s11)
12. Dissolution profiles for 2ME and two series of binary products of 2ME and CDs (p. s12)

1. PXRD traces and single crystal XRD data confirming complex formation between β-CD and 2ME.

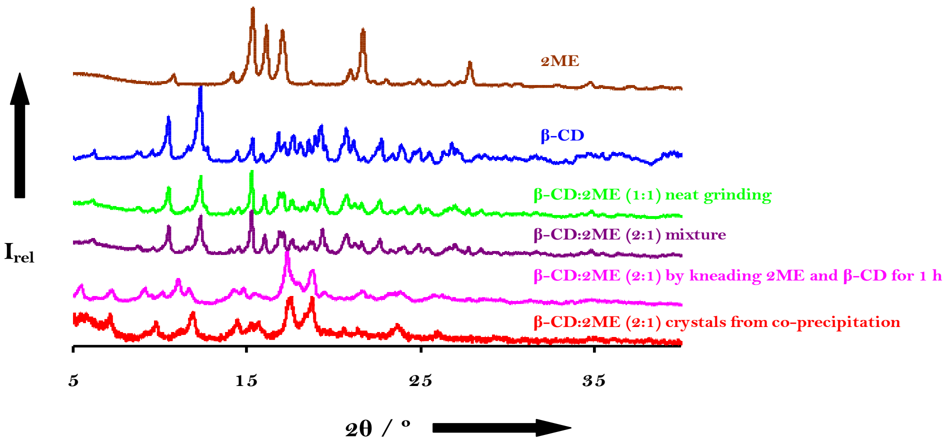

Figure S1: Experimental PXRD patterns of 2ME, β-CD and various preparations containing the two components.

Interpretation: The traces labelled β-CD–2ME (1:1) neat grinding and β-CD–2ME (2:1) mixture do not indicate complex formation as they contain peaks from both of the starting materials. Instead, a distinctly different, common PXRD trace results from the products of kneading and co-precipitation, indicating probable complex formation.

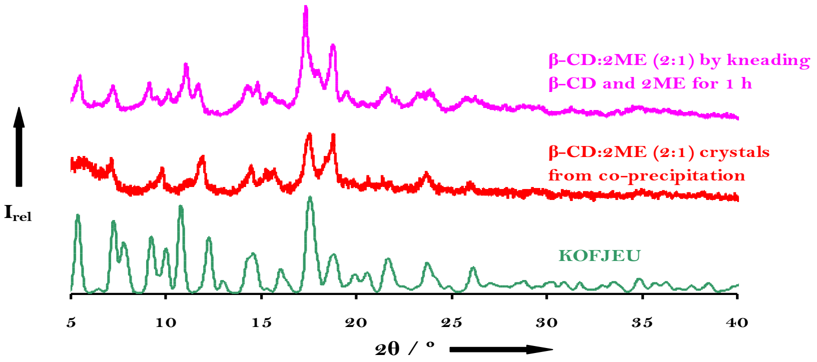

Figure S2: PXRD patterns confirming the isostructurality of the putative complex formed between β-CD and 2ME and the inclusion complex β-cyclodextrin 4-*tert*-butylbenzyl alcohol decahydrate [KOFJEU, space group $C222_1$, a = 19.196(7), b = 24.393(6), c = 32.808(9) Å at room temp. (283–303 °C)].

Interpretation: Close similarities between the PXRD trace for complex KOFJEU and those for the putative complex between β-CD and 2ME suggested that these phases are isostructural.
Subsequent investigation of the single crystals obtained by co-precipitation of β-CD and 2ME were found to belong to the space group $C222_1$, with a = 19.376(2), b = 24.053(2), c = 32.412(2) Å at −160 °C.

2. Relevant PXRD traces, DSC traces and FTIR spectra confirming complex Formation between the hosts RAMEB and HPBCD and the guest 2ME

Descriptions and interpretations of the figures below appear in the manuscript.

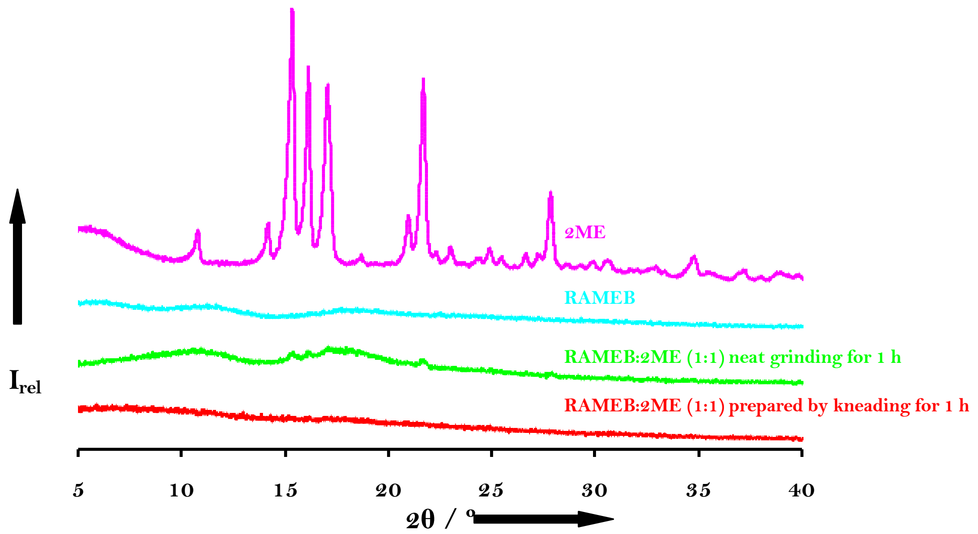

Figure S3: Experimental PXRD patterns of 2ME, RAMEB and various preparations containing the two components.

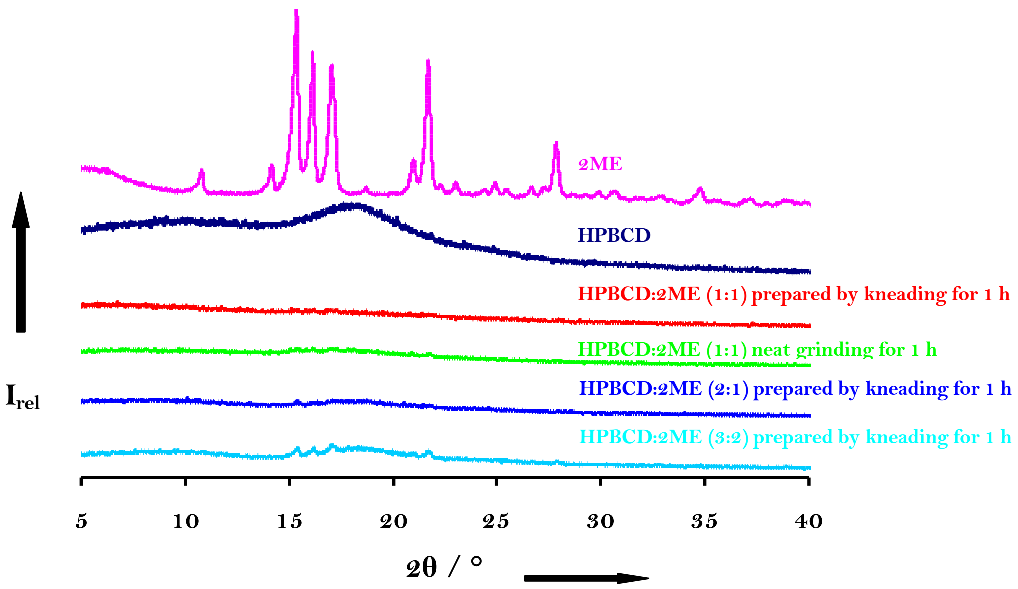

Figure S4: Experimental PXRD patterns of 2ME, HPBCD and various preparations containing the two components.

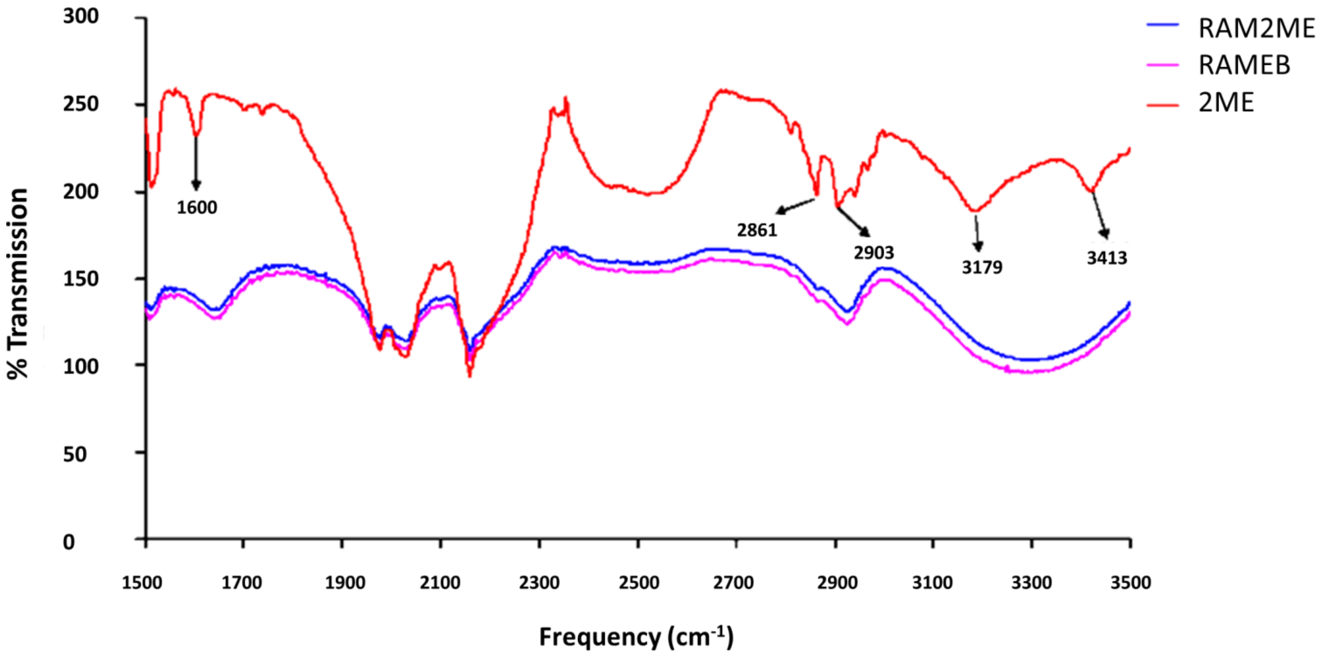

Figure S5: FTIR spectra of RAMEB, 2ME and the spectrum of the putative inclusion complex 'RAM2ME'.

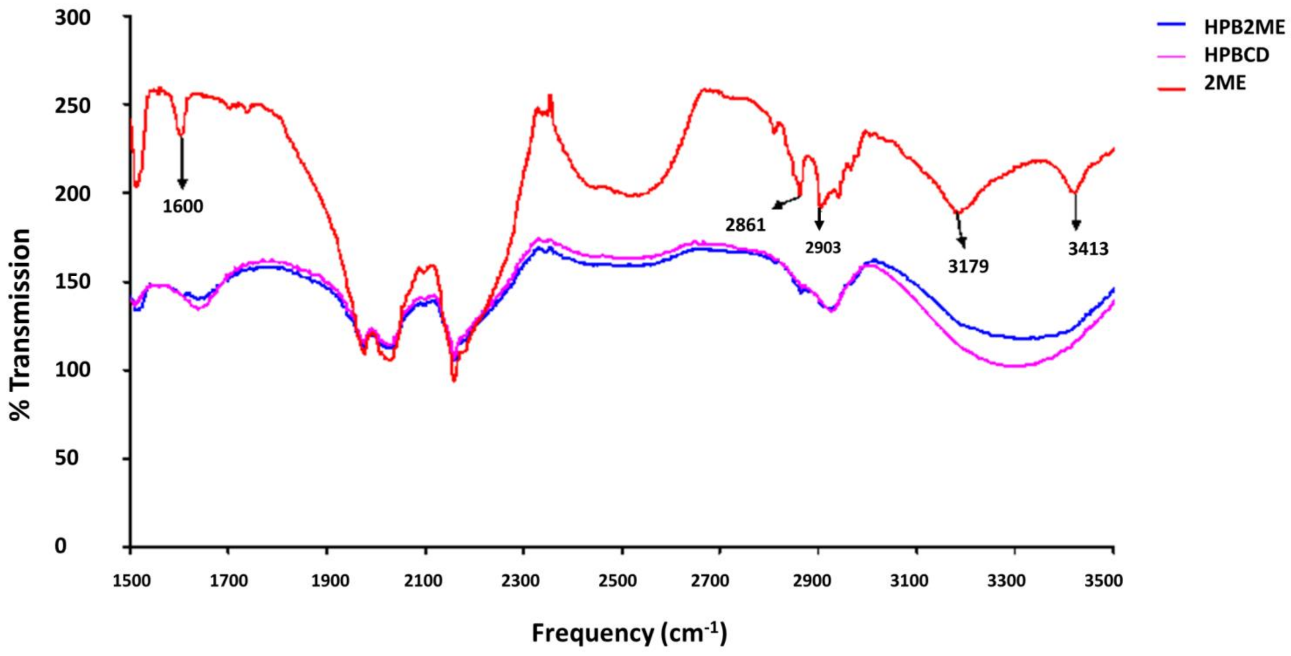

Figure S6: FTIR spectra of HPBCD, 2ME and the spectrum of the putative inclusion complex 'HPB2ME'.

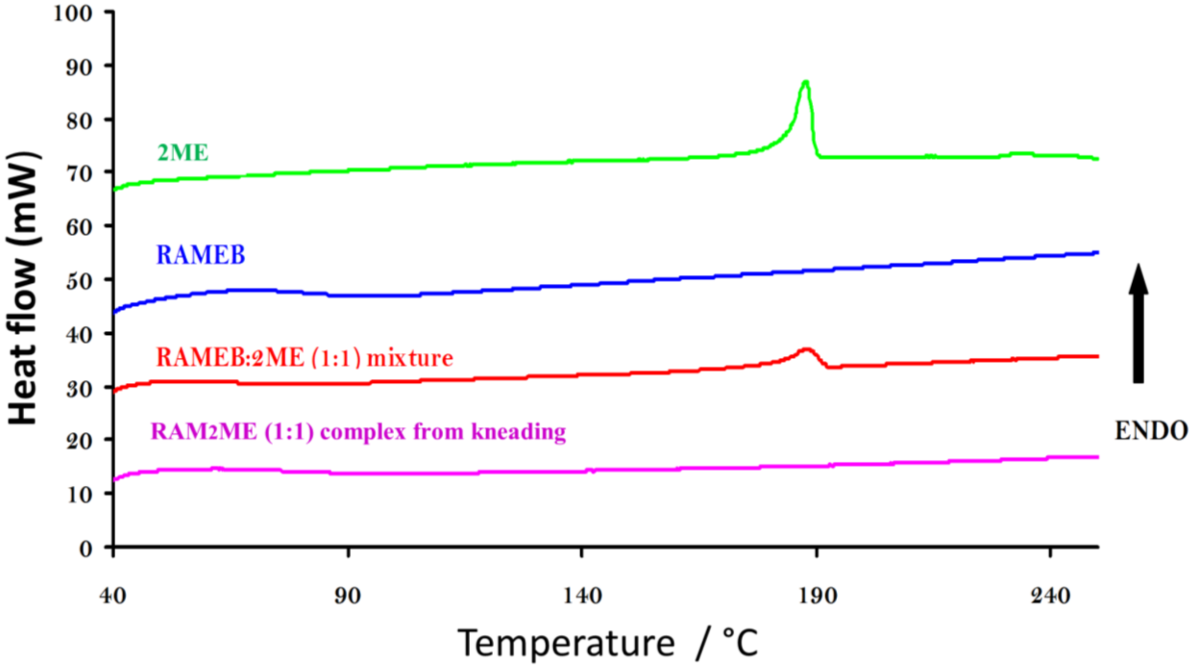

Figure S7: DSC traces for 2ME, RAMEB, their physical mixture and the putative inclusion complex.

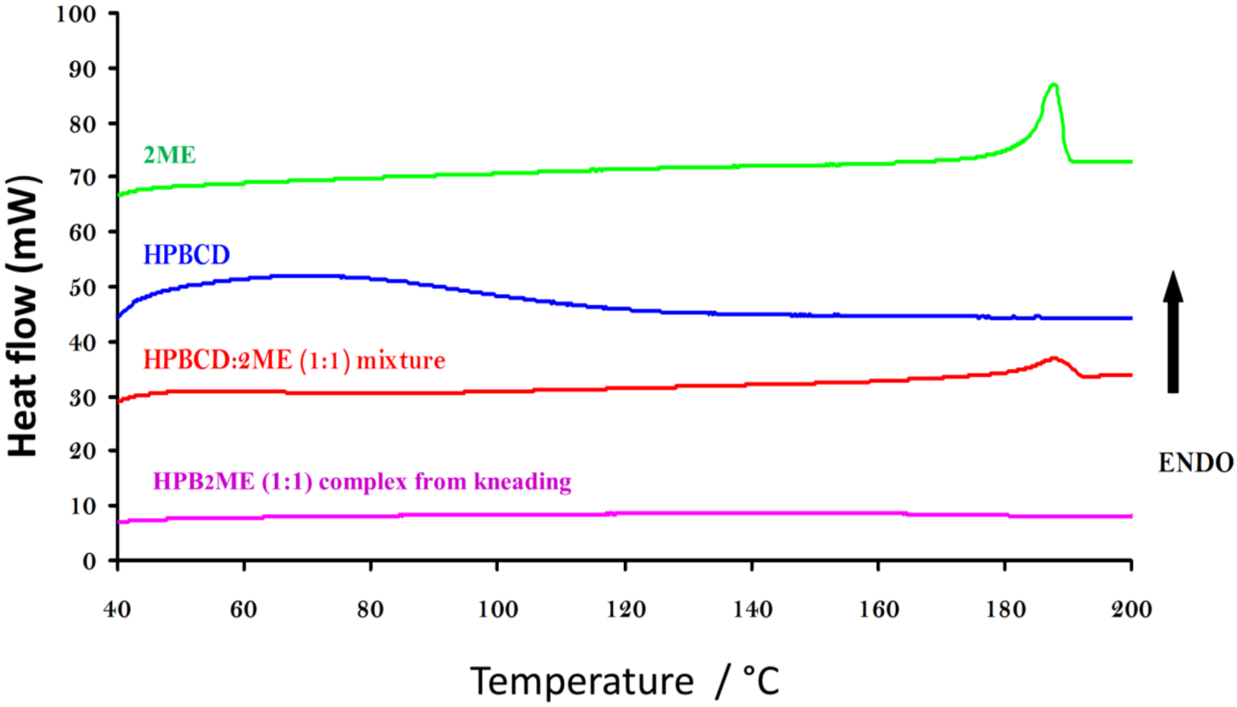

Figure S8: DSC traces for 2ME, HPBCD, their physical mixture and the putative inclusion complex.

3. Thermoanalytical characterization of the DIMEB·2ME and TRIMEB·2ME inclusion complexes

Descriptions and interpretations of the figures below appear in the manuscript.

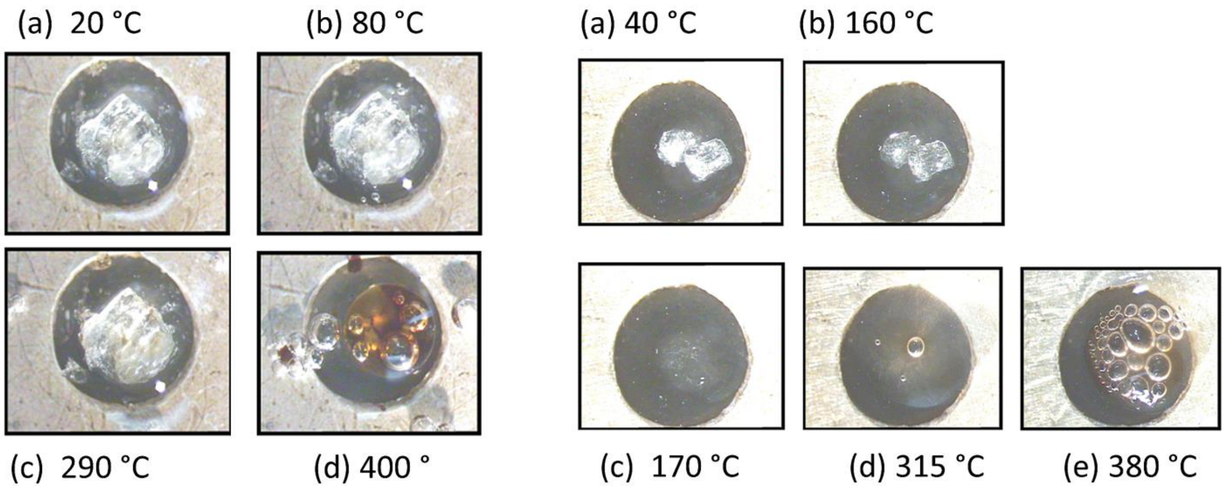

Figure S9: Hot stage micrographs for DIMEB·2ME (left) and TRIMEB·2ME (right).

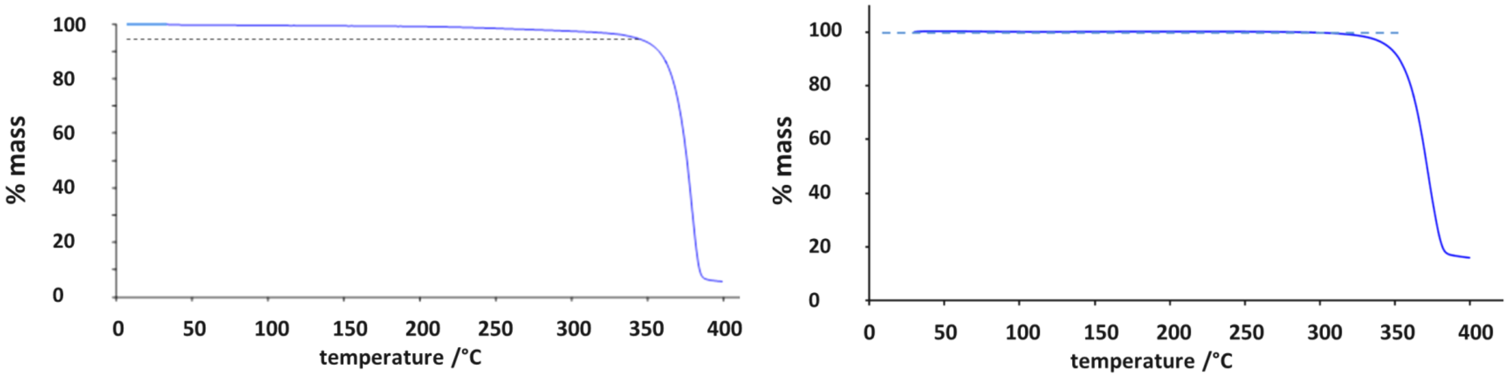

Figure S10: TGA traces for DIMEB·2ME (left) and TRIMEB·2ME (right).

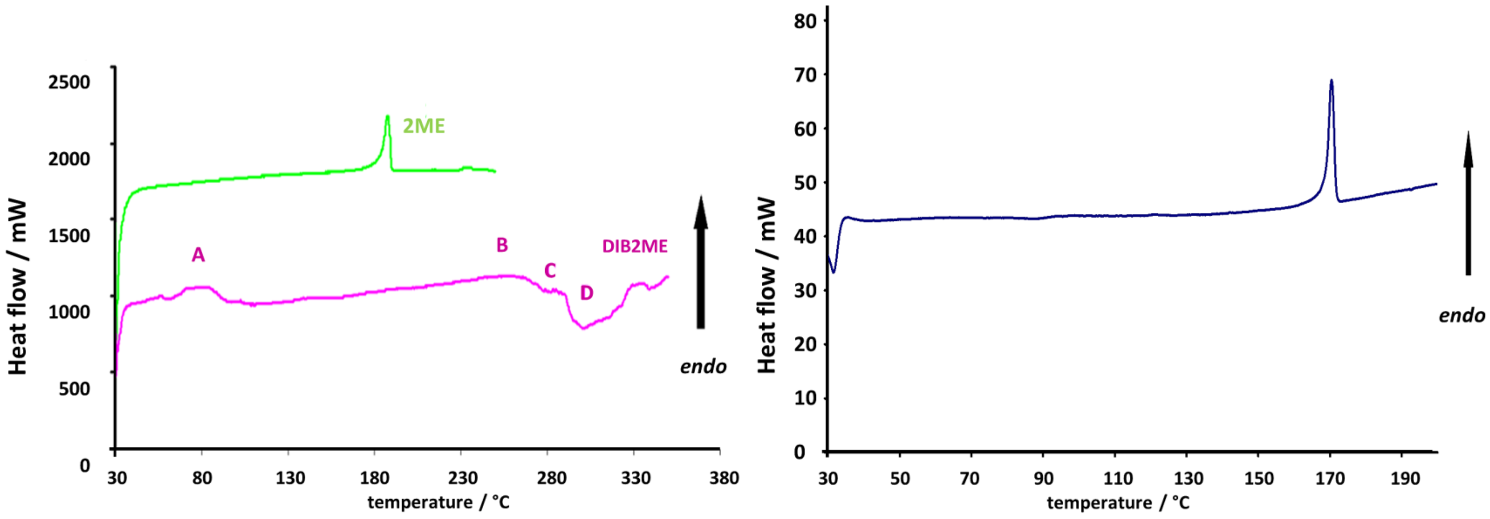

Figure S11: DSC traces for DIMEB·2ME (left) and TRIMEB·2ME (right).

4. Geometrical data for the host DIMEB in the inclusion complex DIMEB·2ME

TABLE S1: Geometrical data for the host molecule DIMEB in the complex DIMEB·2ME

Residue	*r* (Å)	*D* (Å)	*a* (°)	*d* (°)	ϕ (°)	D_3 (Å)	α (Å)	τ_2 (°)
G1	5.023	4.383	129.6	3.8	117.8	2.814(5)	0.063(3)	10.9(2)
G2	5.119	4.372	126.6	2.1	119.1	2.771(5)	-0.097(2)	14.2(2)
G3	5.031	4.404	129.6	-5.6	117.8	2.798(6)	0.016(2)	9.5(4)
G4	5.029	4.396	128.6	4.0	118.3	2.803(4)	0.082(3)	13.6(3)
G5	5.065	4.358	128.7	-1.9	117.7	2.878(5)	-0.075(2)	12.0(1)
G6	5.100	4.419	127.4	3.6	118.6	2.790(6)	-0.001(2)	12.1(2)
G7	5.024	4.387	129.1	-6.0	118.6	2.883(6)	0.011(2)	8.8(4)

**These parameters are defined as follows:
r, the distance of each O4 atom from the centroid of the O4-polygon;
D, the glycosidic O4(*n*)···O4(*n*+1) distance;
a, the O4(*n*−1)···O4(*n*)···O4(*n*+1) angle;
d, the O4(*n*)···O4(*n*+1)···O4(*n*+2)···O4(*n*+3) torsion angle;
ϕ, the intersaccharidic angle C1(*n*−1)–O4(*n*)–C4(*n*)
D_3, the O3(*n*)···O2(*n*+1) intra-ring distance;
α, the deviation of each O4 atom from the mean O4-plane;
τ_2, tilt angle: the angle between the plane containing the atoms O4(*n*), C4(*n*), C1(*n*) and O4(*n*+1) of a given glucose ring and the mean O4-plane.

5. Molecular overlay of 2ME molecules (uncomplexed and complexed with DIMEB)

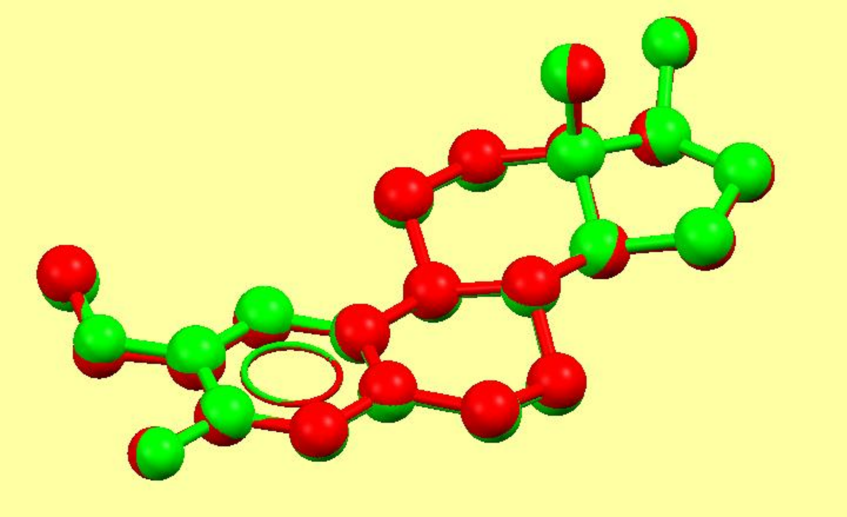

Figure S12: Overlay of the 2ME molecules in the crystal structure of 2ME (red) and encapsulated in the host molecule in the complex DIMEB·2ME (green). The r.m.s.d of the least-squares fit is 0.084 Å, with a maximum deviation of 0.202 Å.

6. Additional hydrogen bond data for TRIMEB·2ME (Table S2)

Hydrogen bonds based on numbering in Figure 2	Atom labels	O···O Distance (Å)
1···2	O22···O1^{i}	2.598(9)
2···3	O1^{i}···O6G2^{i}	2.736(9)
1···4	O22···O3^{ii}	2.674(8)
2···5	O1^{i}···O2^{ii}	2.951(11)
4···5	O3^{ii} ···O2^{ii}	2. 885(10)
5···6	O2^{ii}···O3G4^{ii}	2.759(7)
4···7	O3^{ii}···O3G7^{iii}	2.780(8)
5···8	O2^{ii} ···O8^{iv}	2.623(17)
8···9	O8^{iv}···O6^{iv}	2.647*
8···10	O8^{iv}···O7^{iv}	2.812*
11···12	O6···O8	2.647*
12···13	O8···O7	2.812*
11···15	O6···O9	3.042*
15···16	O9···O5	2.951*

Symmetry operators: (i) *2−x, −1/2+y, −z*; (ii) *1+x, y, z*; (iii) *x, y, −1+z*; (iv) *1+x, y, −1+z.*

NOTE: In Figure 2e, water oxygen atoms labelled 8, 9, 10 are the symmetry-related counterparts of 11, 12 and 13 respectively, the former set being located at (iv) *1+x, y, −1+z* and the latter belonging to the asymmetric unit (symmetry operator *x*, *y*, *z*).

*e.s.d.s not calculated by PLATON due to low site-occupancy of atoms involved.

7. Simulated X-ray photographs for the TRIMEB·2ME complex

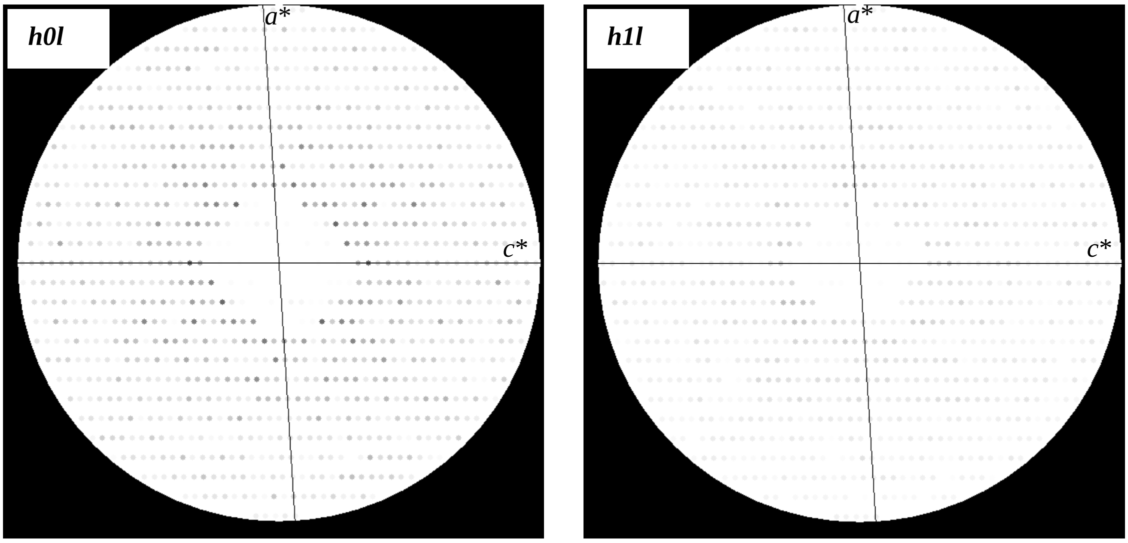

Figure S13: Comparison of representative reciprocal lattice layers of reflections of type *hkl* with $k = 2n$ and $k = 2n+1$ showing typical alternation (strong/weak) in average intensities, indicating the presence of a pseudo-cell with $b' \approx b/2$ in the crystal structure of TRIMEB·2ME. (Images created using program LAYER: L. J. Barbour. LAYER – A computer program for the graphic display of intensity data as simulated precession photographs. *J. Appl. Cryst*., **1999**, 32, 351).

8. Overlay of host molecules A and B in the TRIMEB·2ME complex

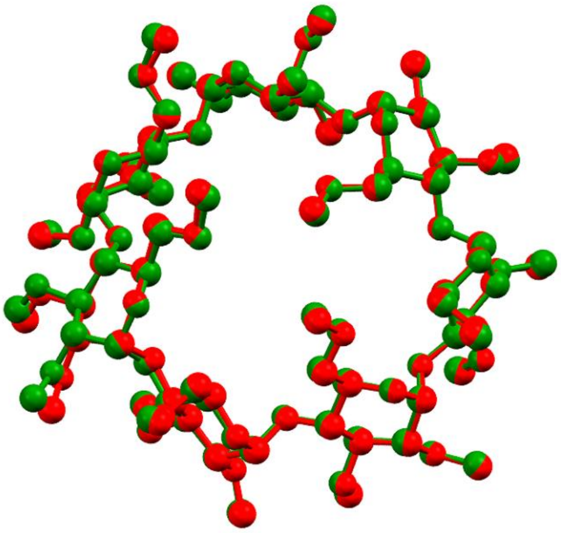

Figure S14: Overlay of the host TRIMEB molecules in complex units A (green) and B (red) of the TRIMEB·2ME crystal (RMSD = 0.157 Å, max. deviation between two equivalent atoms = 0.649 Å).

9. Overlay of host molecules C and D in the TRIMEB·2ME complex

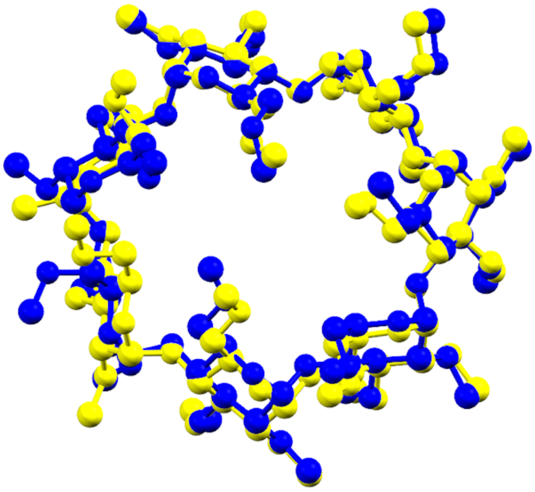

Figure S15: Overlay of the host TRIMEB molecules in complex units C (blue) and D (yellow) of the TRIMEB·2ME crystal (RMSD = 0.819 Å, max. deviation between two equivalent atoms = 3.417 Å).

10. Geometrical parameters for the host molecules A-D in the TRIMEB·2ME complex

TABLE S3: Geometrical data for the four independent TRIMEB host molecules in the complex TRIMEB·2ME

DATA* FOR HOST MOLECULES A AND B								
**Residue**	***r*** **(Å)**	***D*** **(Å)**	***a*** **(°)**	***d*** **(°)**	**ϕ (°)**	**D_3 (Å)**	**α (Å)**	**τ_2 (°)**
A1	5.261	4.497	118.4	-13.7	119.5	3.908	-0.333(3)	10.2(3)
A2	4.733	4.427	138.0	-10.7	113.7	3.410	0.413(3)	25.1(3)
A3	5.248	4.351	120.4	7.8	117.4	3.466	0.113(3)	15.8(3)
A4	5.042	4.558	131.5	14.2	117.0	3.306	-0.376(3)	43.2(2)
A5	5.147	4.447	125.7	-20.3	116.5	3.505	-0.039(3)	16.2(3)
A6	5.081	4.526	124.9	-1.1	117.6	3.498	0.445(3)	9.1(3)
A7	4.904	4.117	135.5	23.8	115.9	3.472	-0.224(3)	50.8(2)
**Residue**	***r*** **(Å)**	***D*** **(Å)**	***a*** **(°)**	***d*** **(°)**	**ϕ (°)**	**D_3 (Å)**	**α (Å)**	**τ_2 (°)**
B7	5.091	4.440	123.4	-12.8	118.8	3.513	-0.391(3)	11.0(4)
B1	4.821	4.399	135.1	-10.5	114.4	3.447	0.406(3)	23.8(3)
B2	5.289	4.378	120.3	5.8	117.6	3.490	0.142(3)	15.3(3)
B3	5.003	4.570	132.1	15.5	116.5	3.327	-0.359(3)	43.7(2)
B4	5.109	4.487	126.9	-17.4	117.2	3.493	-0.087(3)	17.9(3)
B5	5.183	4.509	123.4	-6.2	117.6	3.549	0.461(3)	9.3(3)
B6	4.967	4.168	133.0	26.2	116.2	3.411	-0.173(3)	45.4(2)

TABLE S3: cont'd.

**DATA* FOR HOST MOLECULES C AND D**								
**Residue**	***r* (Å)**	***D* (Å)**	***a* (°)**	***d* (°)**	**ϕ (°)**	**D_3 (Å)**	**α (Å)**	**τ_2 (°)**
C1	4.959	4.483	131.8	0.2	115.3	3.219	-0.309(3)	28.8(3)
C2	4.773	4.497	133.2	10.6	116.1	3.258	0.201(3)	24.0(2)
C3	5.235	4.309	126.8	0.9	117.2	3.237	0.225(3)	31.2(2)
C4	5.449	4.452	117.8	-20.5	118.9	3.662	-0.272(3)	-5.8(4)
C5	4.612	4.508	139.8	15.2	115.4	3.236	-0.151(3)	35.2(3)
C6	4.983	4.353	128.7	5.1	118.4	3.191	0.407(3)	31.4(3)
C7	5.506	4.392	118.1	-15.3	119.4	3.476	-0.100(4)	3.6(3)
**Residue**	***r* (Å)**	***D* (Å)**	***a* (°)**	***d* (°)**	**ϕ (°)**	**D_3 (Å)**	**α (Å)**	**τ_2 (°)**
D1	5.130	4.555	127.3	-10.4	117.5	3.366	-0.028(4)	22.5(4)
D2	4.996	4.511	130.9	5.9	117.2	3.479	-0.095(3)	21.2(2)
D3	5.214	4.299	127.4	8.1	116.6	3.278	0.180(3)	35.7(2)
D4	5.261	4.440	122.5	-15.3	117.6	3.577	-0.029(3)	-5.4(4)
D5	4.706	4.397	139.9	9.4	114.0	3.505	-0.191(3)	40.1(3)
D6	5.239	4.436	122.1	-4.3	117.3	3.477	0.184(3)	20.3(3)
D7	5.227	4.478	128.3	5.9	115.7	3.364	-0.021(4)	24.1(3)

* These parameters are defined as follows:
r, the distance of each O4 atom from the centroid of the O4-polygon;
D, the glycosidic O4(*n*)···O4(*n*+1) distance;
a, the O4(n-1)···O4(*n*)···O4(*n*+1) angle;
d, the O4(n)···O4(*n*+1)···O4(*n*+2)···O4(*n*+3) torsion angle;
ϕ, the intersaccharidic angle C1(*n*-1)–O4(*n*)–C4(*n*)
D_3, the O3(*n*)···O2(*n*−1) intra-ring distance;
α, the deviation of each O4 atom from the mean O4-plane;
τ_2, tilt angle: the angle between the plane containing the atoms O4(*n*), C4(*n*), C1(*n*) and O4(*n*+1) of a given glucose ring and the mean O4-plane.

11. Overlay of the 2ME molecules in TRIMEB·2ME complex unit C and the DIMEB·2ME complex

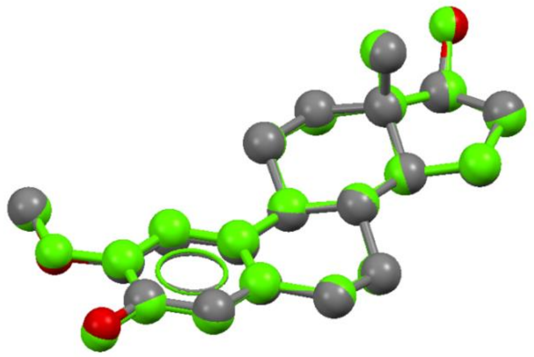

Figure S16: Overlay of the 2ME molecules in the DIMEB·2ME complex (green) and in unit C of the TRIMEB·2ME complex. (R.M.S.D = 0.083 Å with a maximum atomic deviation of 0.161 Å).

12. Dissolution profiles for 2ME and two series of binary products of 2ME and CDs

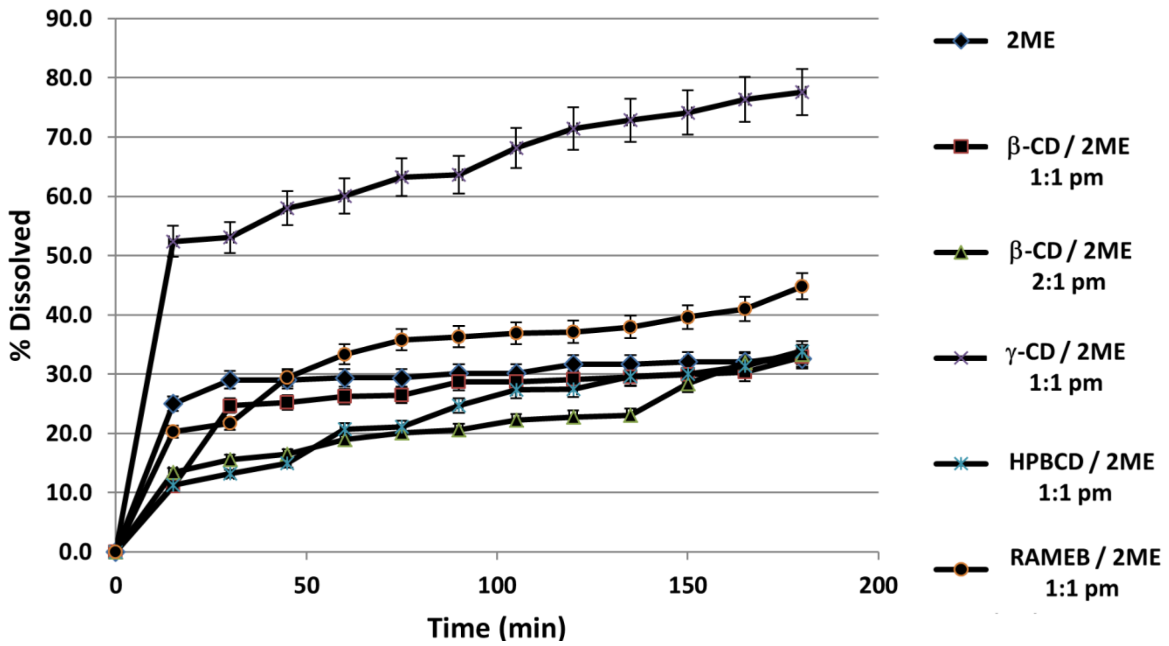

Figure S17: Comparative dissolution profiles for 2ME in physical mixtures (pm) with the native cyclodextrins β- and γ-CD as well as RAMEB and HPBCD.

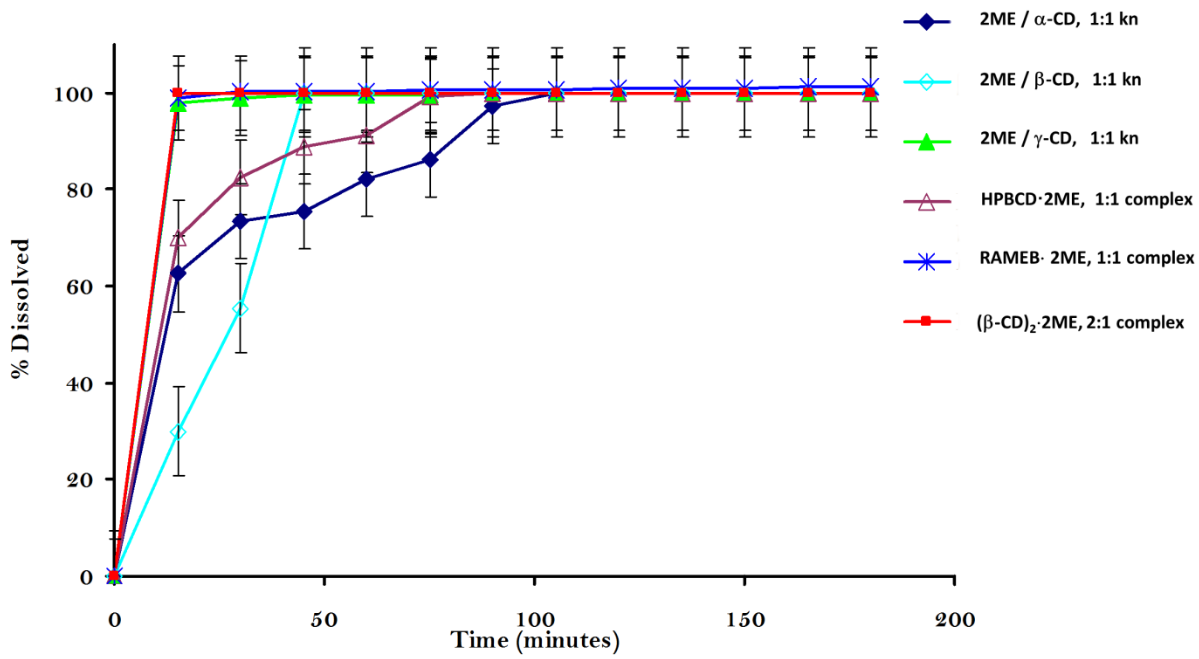

Figure S18: Dissolution profile of the inclusion complex $(\beta\text{-CD})_2\cdot$2ME and those of a series of 2ME/CD samples resulting from kneading (kn) as well as 1:1 inclusion complexes of HPBCD and RAMEB.
